# Supplementary figures and images for: Reconstructing tumor evolutionary histories and clone trees in polynomial-time with SubMARine
Source: PLoS Comput Biol. 2021 Jan 19;17(1):e1008400. doi: 10.1371/journal.pcbi.1008400 (PMC7845980; doi:10.1371/journal.pcbi.1008400)

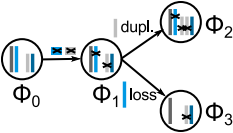

Supplement: S1 Fig — Subclonal frequencies are indicated with ϕ0, …, ϕ3; assuming that there are two samples given, their values could be ϕ0 = (1, 1), ϕ1 = (0.9, 0.8), ϕ2 = (0.5, 0.3), and ϕ3 = (0.4, 0.35). Edges between subclones indicate ancestral relationships, with the germline being an ancestor of all subclones and subclone 1 being the ancestor of subclones 2 and 3. Colorful bars indicate alleles of different segments; here, the two alleles of two segments are shown, with segment 1 having the dark gray and the light blue alleles, and segment 2 having the light gray and dark blue alleles. Two SSMs are assigned to subclone 1, one to the blue allele of segment 1 and one to the gray allele of segment 2. The SSMs are inherited by the descendants of subclone 1. Furthermore, two CNAs are assigned to the subclones, shown as copy number changes. One copy number duplication of the gray allele of segment 2 is assigned to subclone 2, duplicating the SSM lying on it. One copy number loss of the blue allele of segment 1 is assigned to subclone 3, deleting with it the SSM of this segment. (PDF) [file pcbi.1008400.s001.pdf]

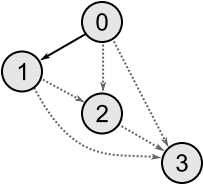

Supplement: S2 Fig — Given t with subclonal frequency matrix ϕ = (1, 0.7, 0.3, 0.2)T, this partial clone tree is its MAR. Six clone trees complete the MAR, however, only five of them are valid. The clone tree in which the germline is a parent of subclones 2 and 3 does not satisfy the sum constraint and hence is not a solution to t. (PDF) [file pcbi.1008400.s002.pdf]

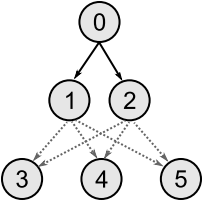

Supplement: S3 Fig — Example of a valid partial clone tree given the subclonal frequency matrix ϕ = ((1.0, 1.0), (0.6, 0.6), (0.4, 0.4), (0.39, 0.37), (0.38, 0.38), (0.37, 0.39))T. Subclones 1 and 2 are definite children of the germline. Subclones 1 and 2 do not have definite children because their ancestral relationships to subclones 3, 4 and 5 are undefined. In a completion without undefined relationships, either subclone 1 or 2 would have to have two definite children. However, given the frequencies in ϕ, subclones 1 and 2 can have only one definite child without violating the generalized sum constraint. Thus, there exists no valid full completion of this valid partial clone tree. (PDF) [file pcbi.1008400.s003.pdf]

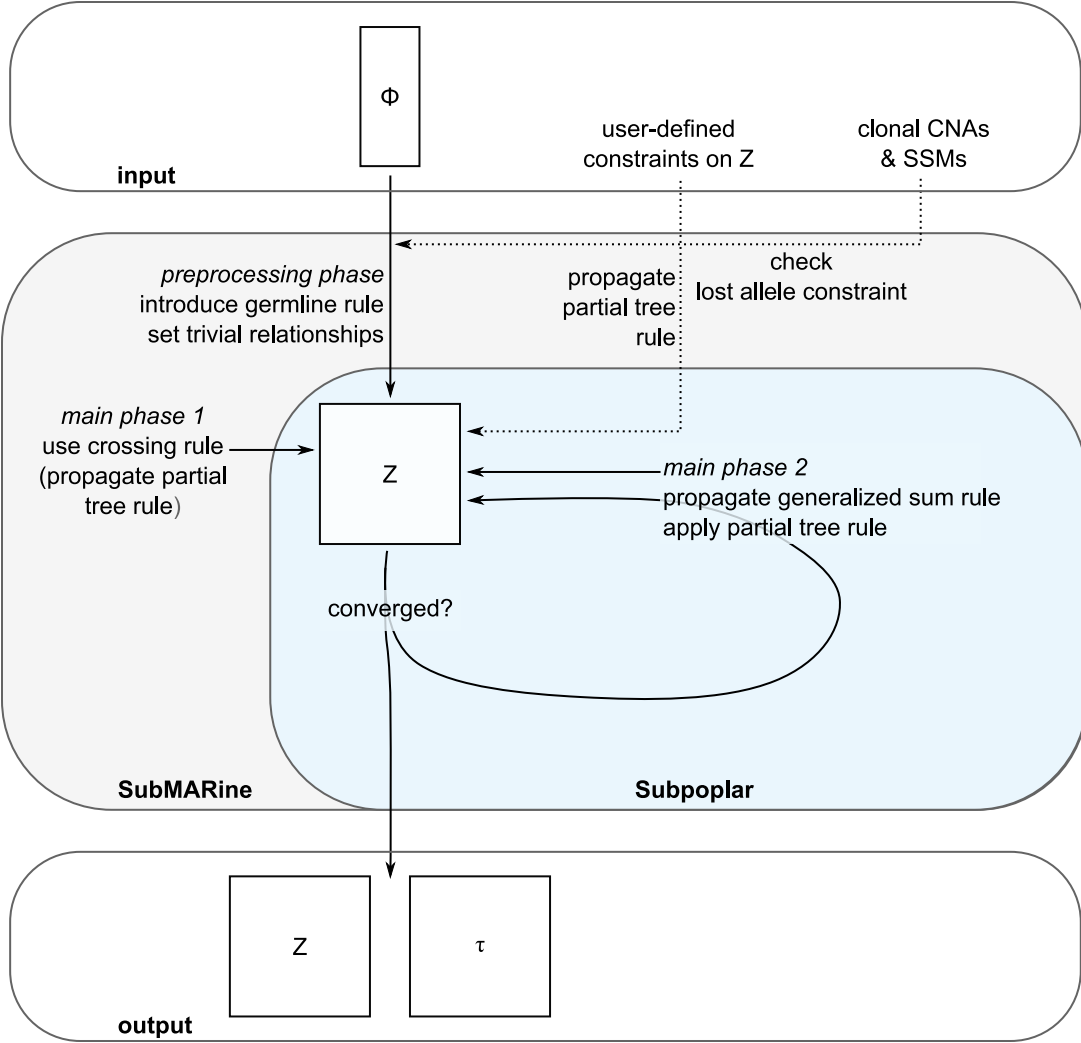

Supplement: S4 Fig — The basic version of SubMARine takes the subclonal frequency matrix ϕ as input to build the ancestry matrix Z. In a preprocessing phase, the germline rule is introduced by setting Z(0, k) = 1 for all k > 0. Also, all trivial relationships are set to 0 (Z(k, k′) = 0 for k′ ≤ k) as a consequence of the generalized sum constraint. Then, the main phase starts by using the crossing rule (Eq (9), Section III.1 in S1 Text), which also follows from the generalized sum constraint. The generalized sum rule itself and the partial tree rule are propagated by using Subpoplar until the ancestry matrix converged and no more relationships can be defined. Then, SubMARine outputs the ancestry matrix Z together with the possible parent matrix τ, created by Subpoplar. When the user defines additional constraints on Z, these are also input to SubMARine. They are applied after the preprocessing phase, followed by a propagation of the partial tree rule. This rule is also propagated now when using the crossing rule. The reason is that with the entries set by the user, Z can contain 1’s in other positions than the first row, possibly requiring updates of undefined relationships. Without user-defined constraints on Z, 1’s in other rows can be set only in Subpoplar, hence the partial tree rule needs to be applied only at that stage. When the user provides clonal CNAs and SSMs as input, the lost allele constraint is checked before starting the preprocessing phase. Whenever a constraint cannot be satisfied, SubMARine terminates and indicates which subclonal relationship led to the conflict. (PDF) [file pcbi.1008400.s004.pdf]

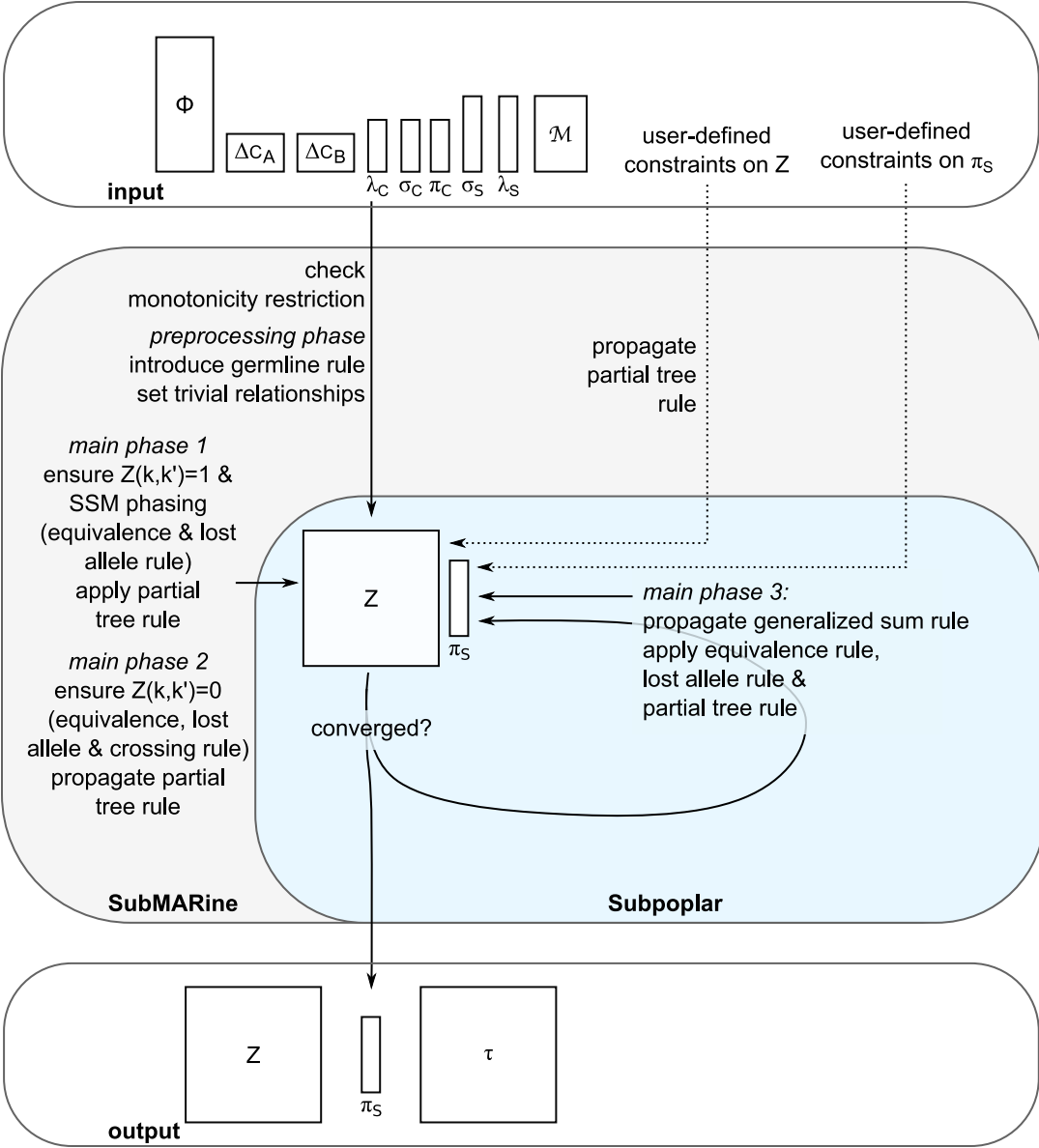

Supplement: S5 Fig — The extended version of SubMARine takes the subclonal frequency matrix ϕ, CNAs as copy number changes in the matrices ΔCA and ΔCB, assigned to subclones, segments and parental alleles in the vectors λc, σc and πc, SSMs assigned to segments and subclones in the vectors σs and λs, and the impact matrix M as input to build the ancestry matrix Z and the SSM phasing vector πs. At first, the monotonicity restriction is checked to hold on the CNAs. Then, in the preprocessing phase, the germline rule is introduced and trivial relationships (Z(k, k′) = 0 for k′ ≤ k) are set. Afterwards, SubMARine starts the main phase, ensuring that the partial tree rule is applied each time a relationship is updated. First, the equivalence rule based on Eq (13) in Section IV.3 in S1 Text is propagated, leading to 1’s in Z, together with those equivalence and lost allele rules that update SSM phasing. Second those equivalence and lost allele rules that lead to 0’s in Z and the crossing rule are used. Third, the general sum rule is propagated with Subpoplar, which also applies the equivalence, lost allele and partial tree rules whenever necessary. The method converges, when no more subclonal relationships and SSM phases can be updated. The output consists of the ancestry matrix Z, the SSM phasing vector πs and the possible parent matrix τ, created by Subpoplar. The user can also define additional constraints on Z and on πs. Both types of constraints are applied after the preprocessing step and before the main phase starts. When user-constraints on Z are set, the partial tree rule is already propagated before the main phase. Whenever a constraint cannot be satisfied, SubMARine terminates and indicates what led to the conflict. (PDF) [file pcbi.1008400.s005.pdf]

Required relationships for datasets with 20 subclones

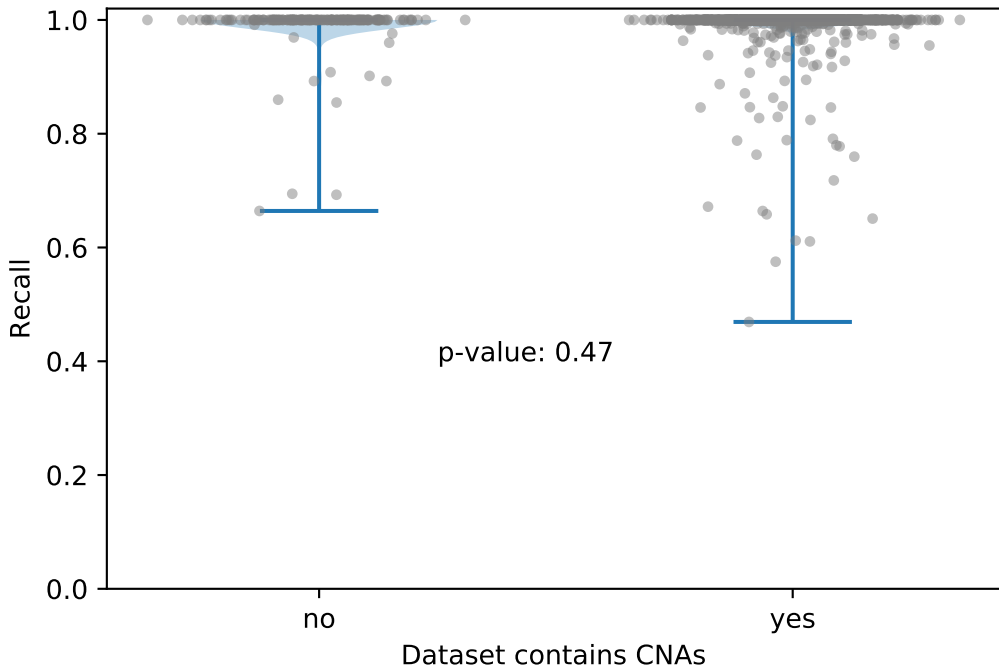

Supplement: S9 Fig — The recall is computed based on the non-trivial ancestral relationships. The p-value is computed with a Mann-Withney U test. The left column contains 193 data points and the right 1775. (PDF) [file pcbi.1008400.s009.pdf]

A

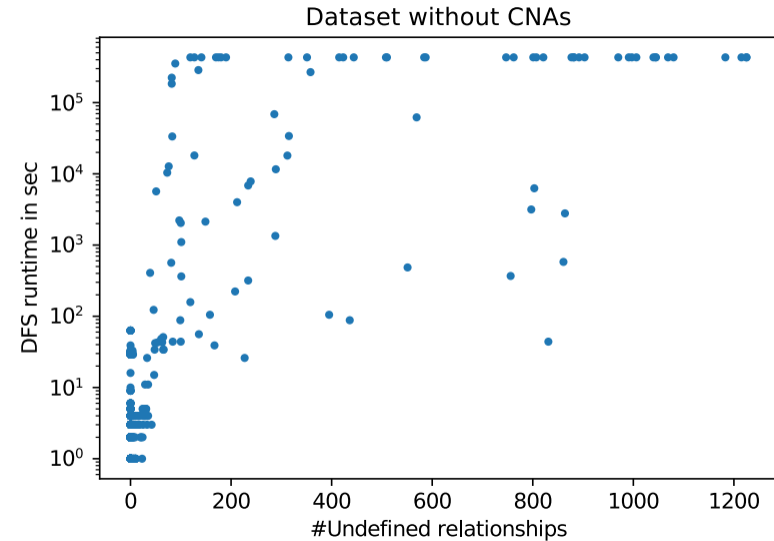

B

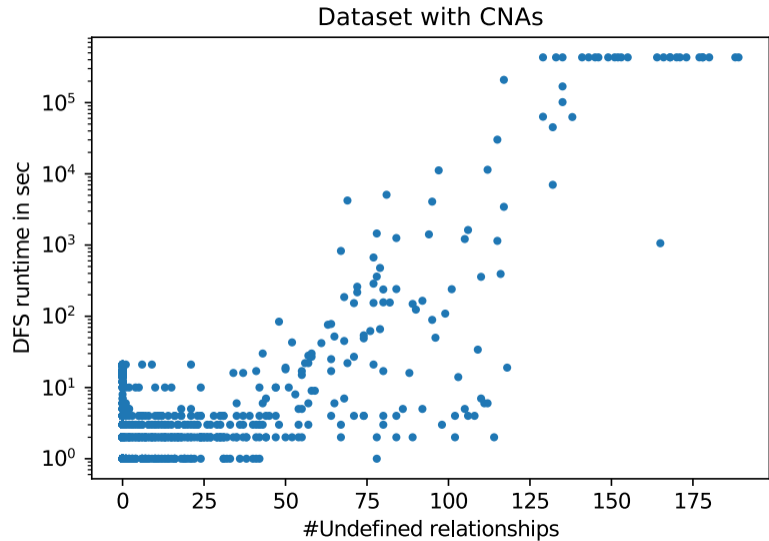

Supplement: S10 Fig — We terminated searches exceeding a maximal runtime of 120 h. We used two versions of the DFS to enumerate clone trees for different subMARs for the dataset without CNAs. The first version is a naïve, recursive one and the second version is an improved, iterative and also faster one, which we provide with SubMARine. Hence, if using the second version to enumerate the clone trees of all subMARs, the overall runtime could be improved. Note that for all subMARs on which the search did not termindate in 120 h, as well as for all subMARs of the dataset with CNAs, we already used the faster version. (PDF) [file pcbi.1008400.s010.pdf]

Distribution among the three different noise buffer statutes

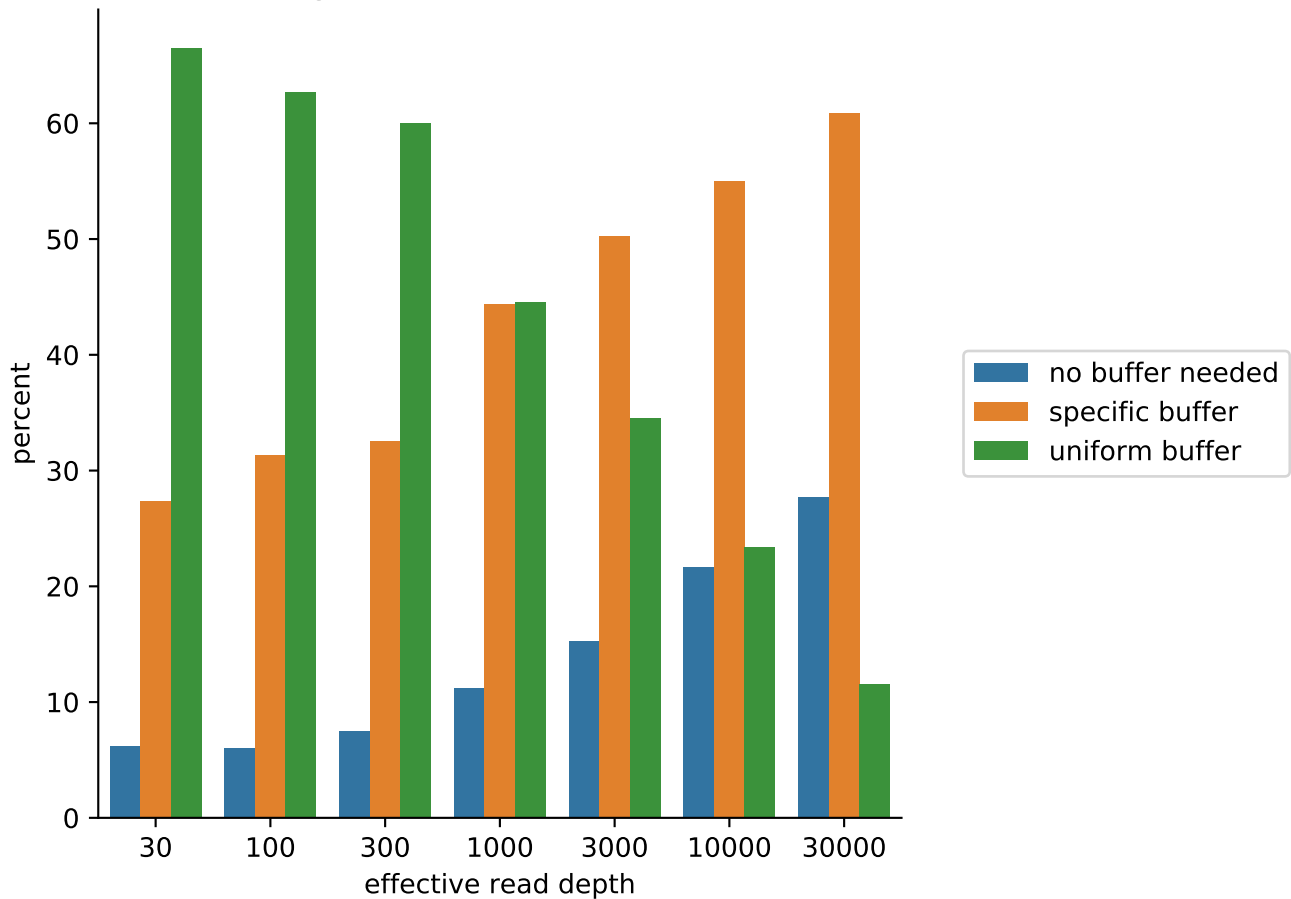

Supplement: S11 Fig — There are three different noise buffer statuses: Either no noise buffer is needed to build a subMAR, the subclone- and sample specific buffer set can be found in polynomial time, or the uniform buffer is used. The datasets with an effective read depth of 300 and 3000 contain 1200 subMARs, all others 600. (PDF) [file pcbi.1008400.s011.pdf]

Proportion of subclones with uncertain parentage  
for effective read depth

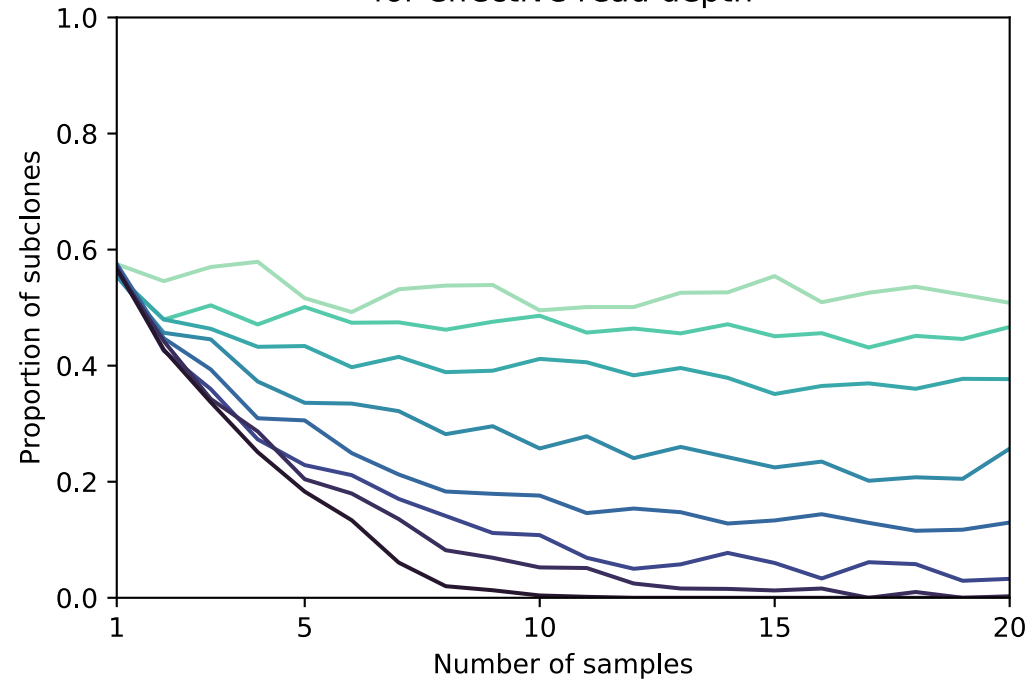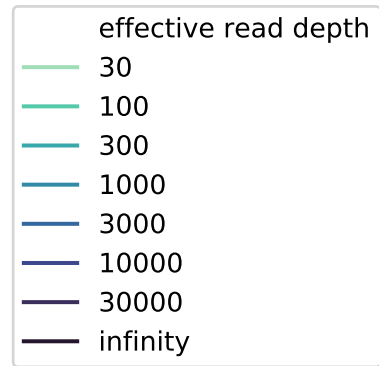

Supplement: S13 Fig — A subclone has uncertain parentage when it has multiple possible parents in the possible parent matrix τ. Line shows mean. The lowest line for an effective read depth of infinity shows the mean uncertain parentage of the corresponding noise-free simulated data. (PDF) [file pcbi.1008400.s013.pdf]

Proportion of subclones with uncertain parentage  
for effective read depth and 5 subclones

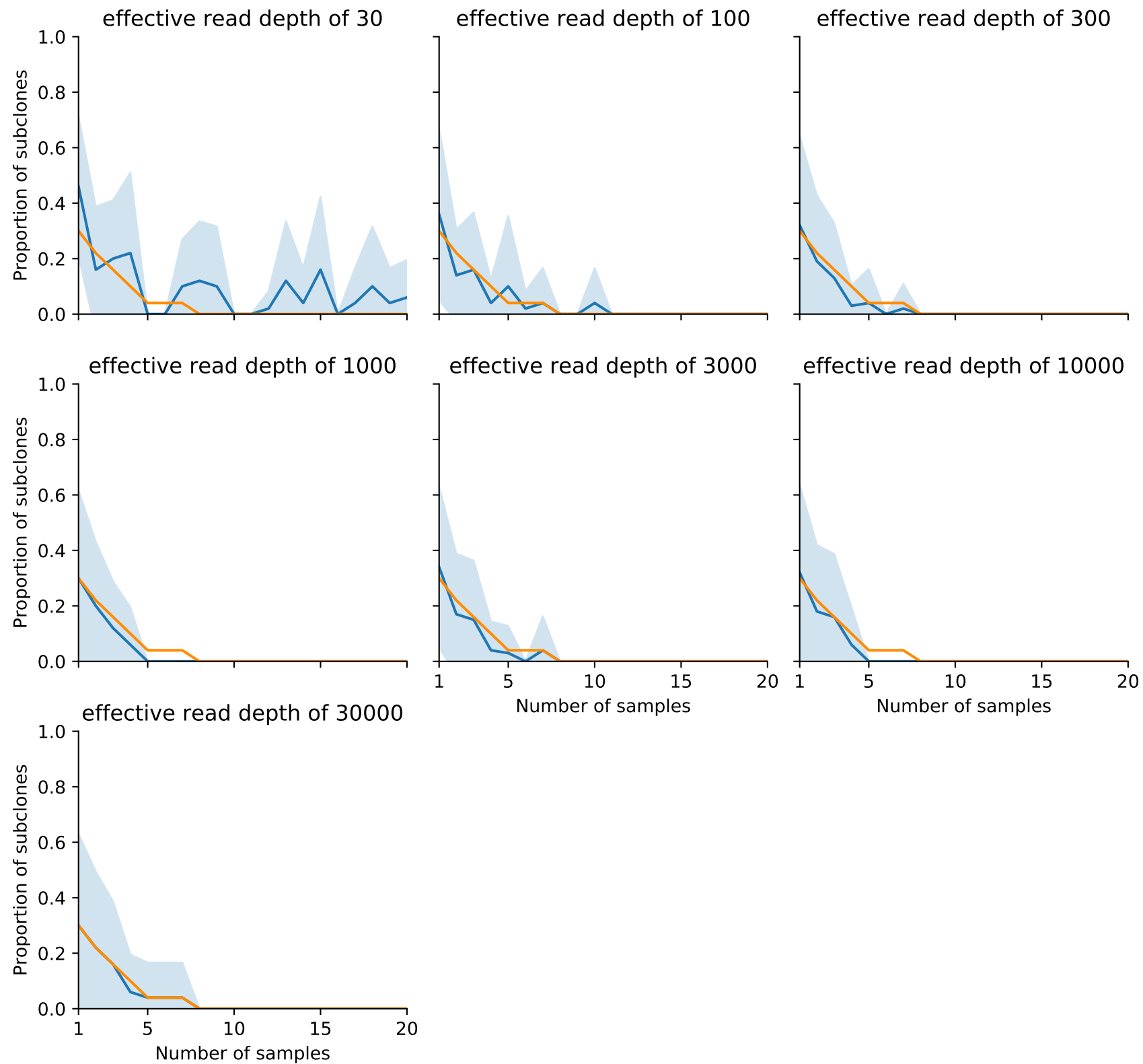

Supplement: S14 Fig — A subclone has uncertain parentage when it has multiple possible parents in the possible parent matrix τ. Blue line shows mean and blue area standard deviation of uncertain parentage on noisy data. Orange line shows mean of corresponding noise-free data. (PDF) [file pcbi.1008400.s014.pdf]

Proportion of subclones with uncertain parentage  
for effective read depth and 20 subclones

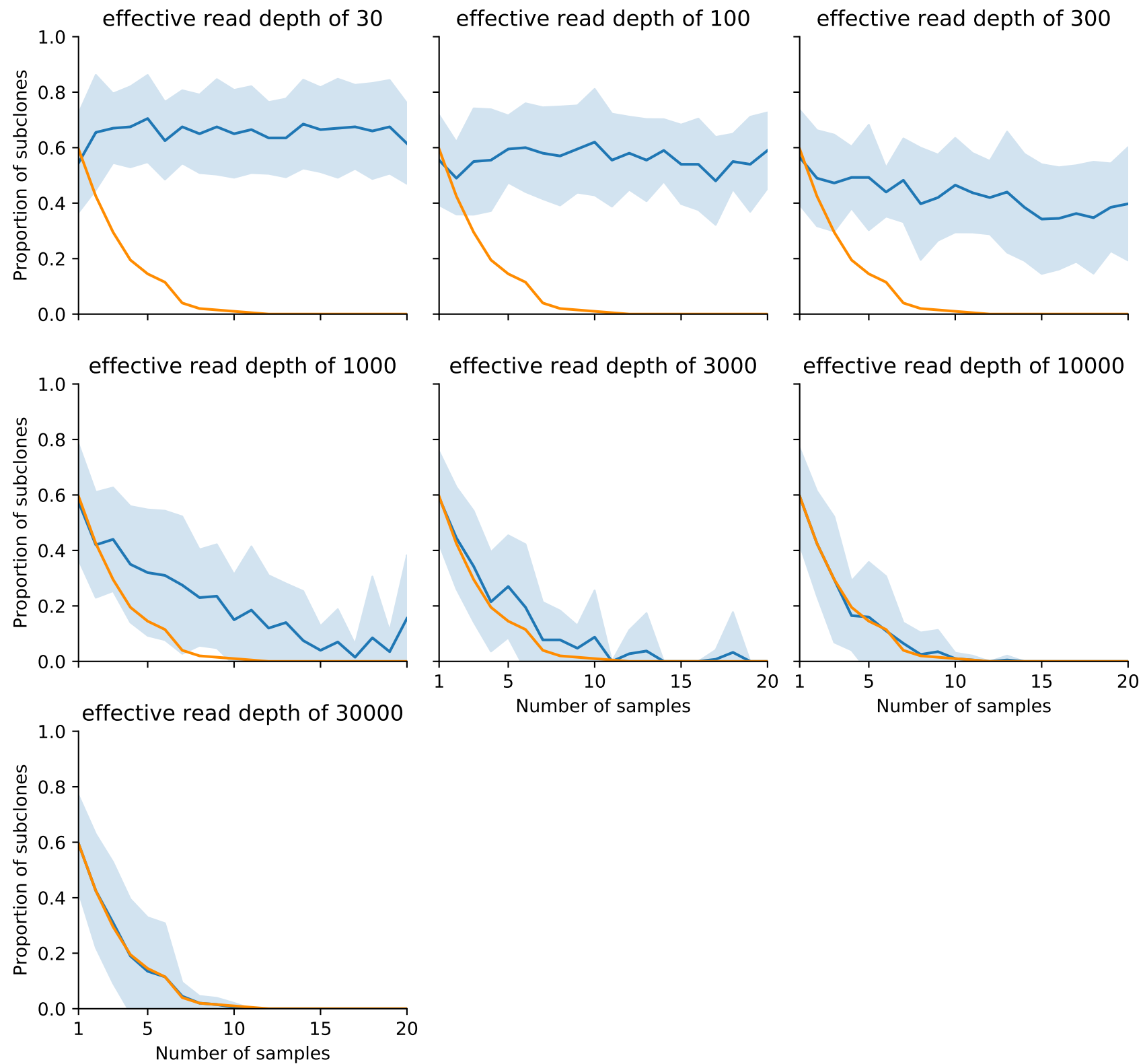

Supplement: S15 Fig — A subclone has uncertain parentage when it has multiple possible parents in the possible parent matrix τ. Blue line shows mean and blue area standard deviation of uncertain parentage on noisy data. Orange line shows mean of corresponding noise-free data. (PDF) [file pcbi.1008400.s015.pdf]

Proportion of subclones with uncertain parentage  
for effective read depth and 50 subclones

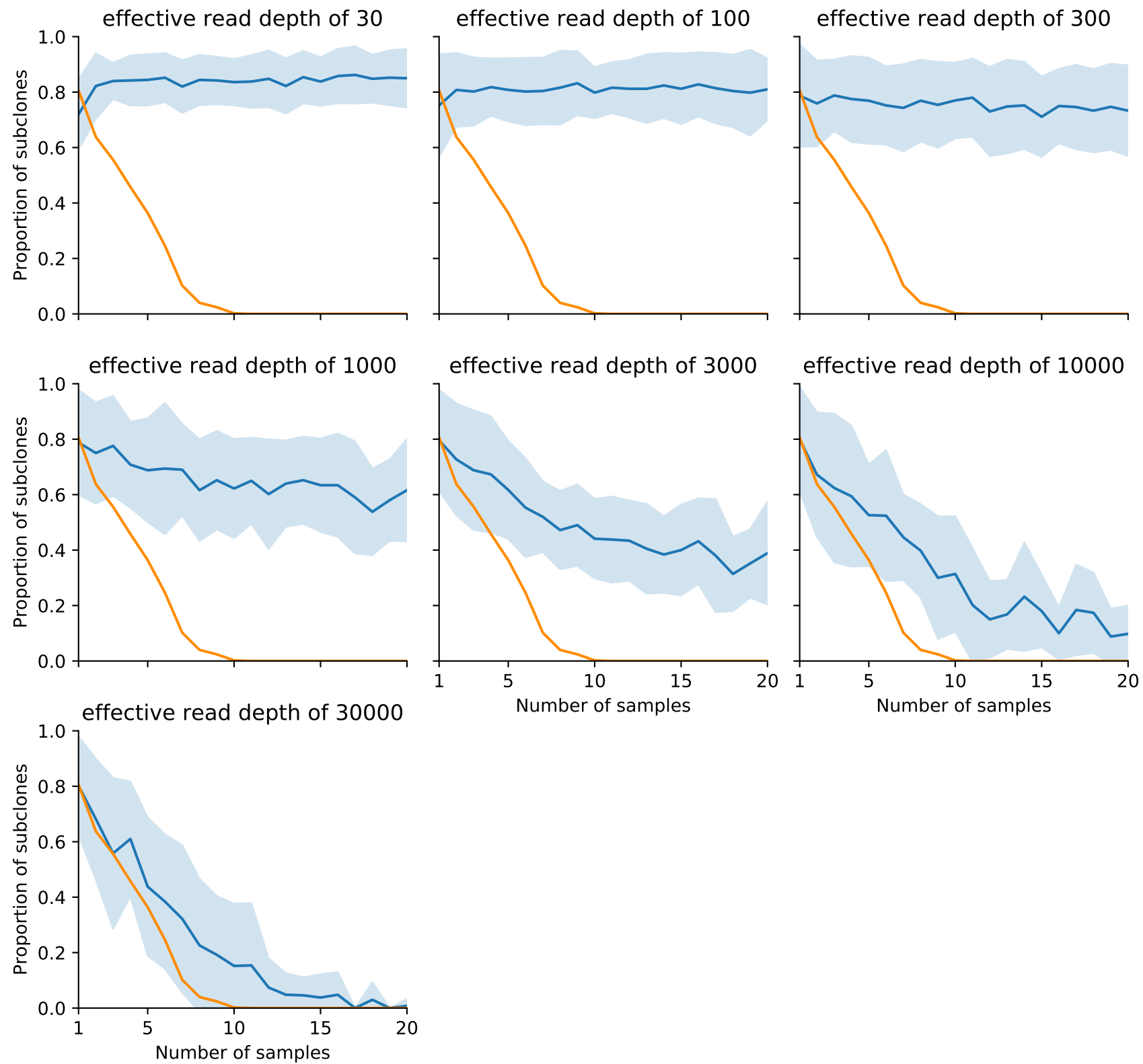

Supplement: S16 Fig — A subclone has uncertain parentage when it has multiple possible parents in the possible parent matrix τ. Blue line shows mean and blue area standard deviation of uncertain parentage on noisy data. Orange line shows mean of corresponding noise-free data. (PDF) [file pcbi.1008400.s016.pdf]

A

Falsely defined error

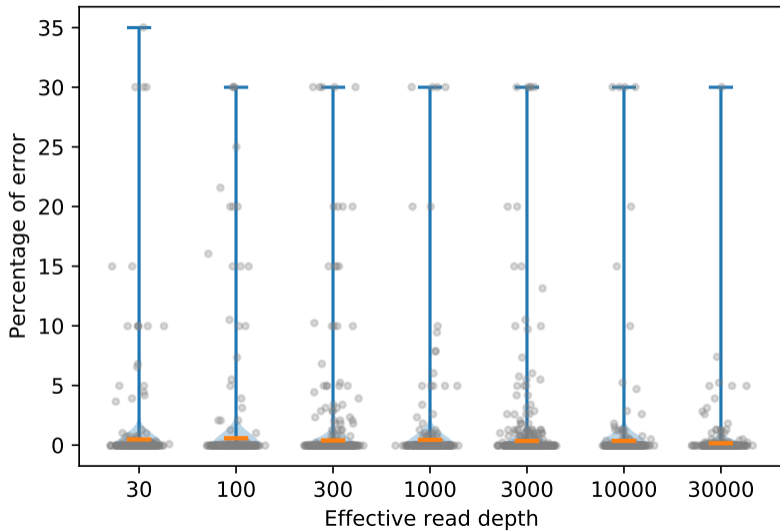

B

Differently defined error

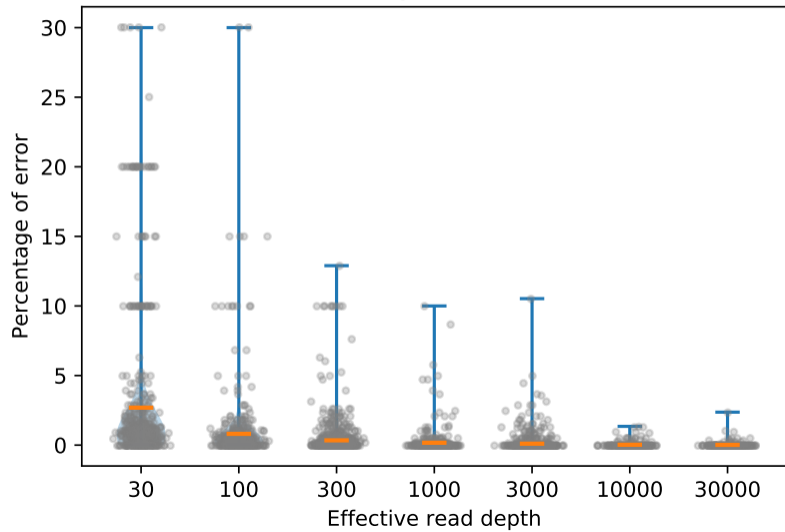

Supplement: S17 Fig — (PDF) [file pcbi.1008400.s017.pdf]

CRUK0011

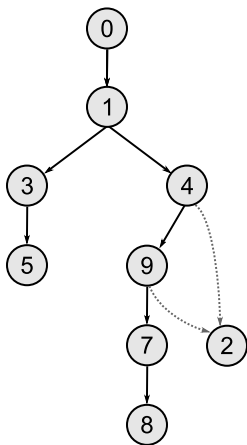

CRUK0037

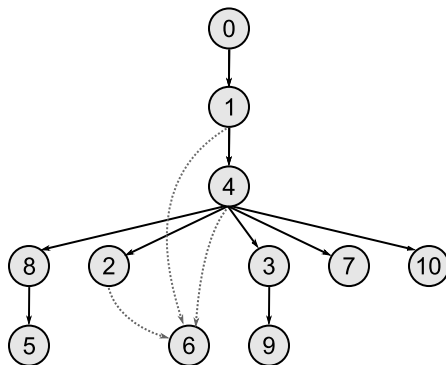

CRUK0046

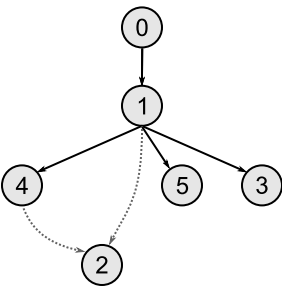

CRUK0094

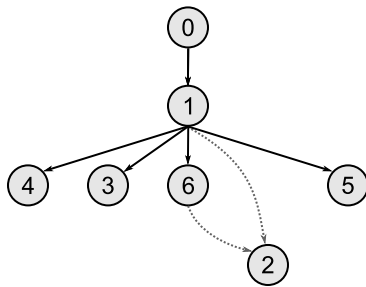

CRUK0099

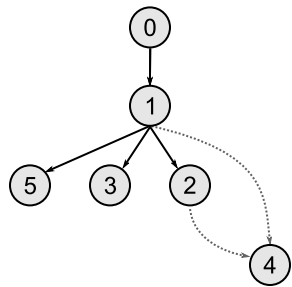

Supplement: S19 Fig — Shown are the subMARs that contain undefined ancestral relationships. They are identical to their MARs. Subclonal indices are taken from the TRACERx mutation clusters. (PDF) [file pcbi.1008400.s019.pdf]

Noise buffers among the TRACERx dataset

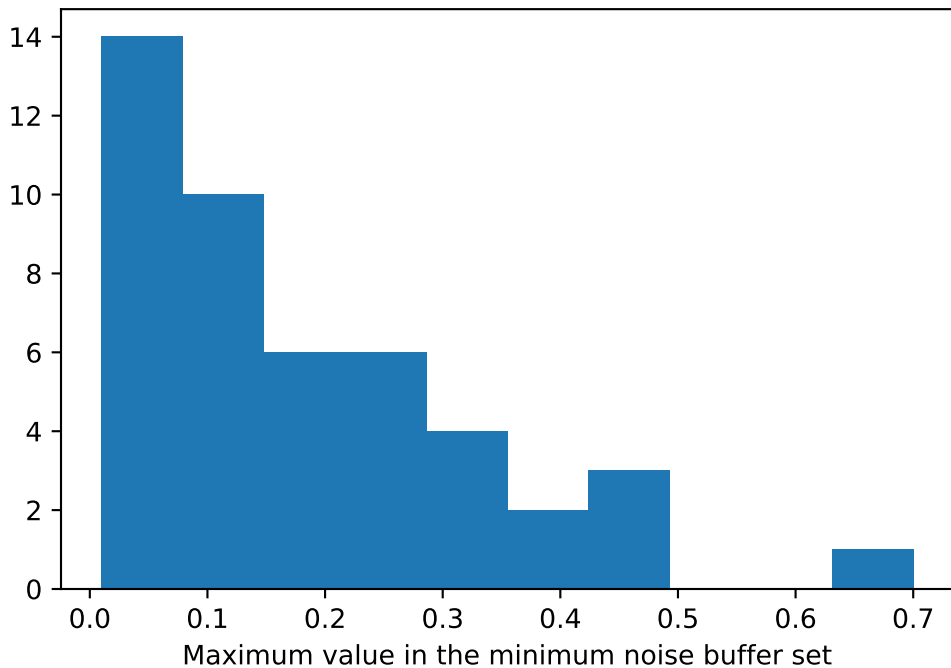

Supplement: S20 Fig — (PDF) [file pcbi.1008400.s020.pdf]

A

| $\phi$ | 0    | 1    | 2    |
|--------|------|------|------|
| 0      | 1.0  | 1.0  | 1.0  |
| 1      | 1.0  | 1.0  | 1.0  |
| 2      | 0    | 0.94 | 0.81 |
| 3      | 0.67 | 0    | 0    |
| 4      | 0.96 | 0    | 0.35 |
| 5      | 0.97 | 0    | 0.89 |
| 6      | 0    | 0.94 | 0.01 |

B

CRUK0078

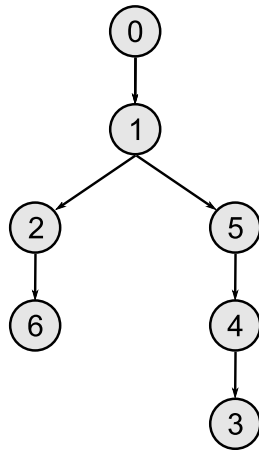

Supplement: S21 Fig — Subclonal indices are taken from the TRACERx mutation clusters. Both subclones 2 and 5 are children of subclone 1. However, they have a subclonal frequency of 0.81 and 0.89, respectively, in sample 2. Hence, a noise buffer of 0.7 is necessary. (PDF) [file pcbi.1008400.s021.pdf]

A

| $\phi$ | 0    | 1    | 2    |
|--------|------|------|------|
| 0      | 1.0  | 1.0  | 1.0  |
| 1      | 0.99 | 0.99 | 0.98 |
| 4      | 0.76 | 0.91 | 0.78 |
| 2      | 0.63 | 0.25 | 0.38 |
| 3      | 0    | 0.35 | 0.05 |

B

CRUK0095

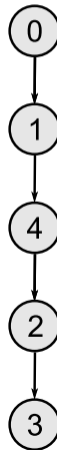

Supplement: S22 Fig — Subclonal indices are taken from the TRACERx mutation clusters. Given the shown subclonal frequencies and the clone tree, the sum constraint is not satisfied because Z(2, 3) = 1 although ϕ(2, 1) < ϕ(3, 1). Hence, CITUP must have inferred other subclonal frequencies. (PDF) [file pcbi.1008400.s022.pdf]

**A22**  
adapted (raw)

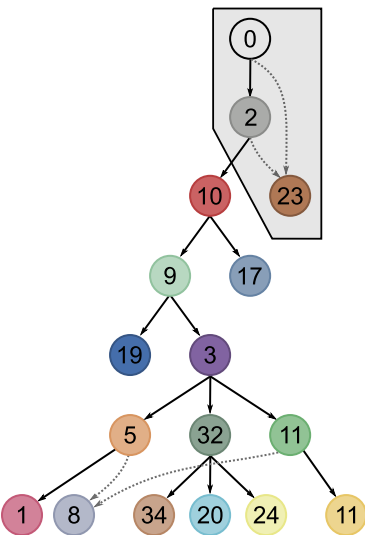

**A24**  
raw & adapted

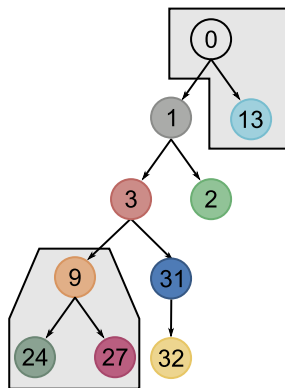

**A29**  
adapted (raw)

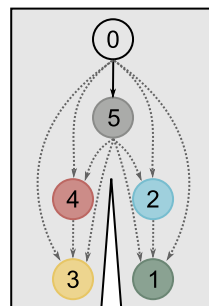

**A31**  
adapted

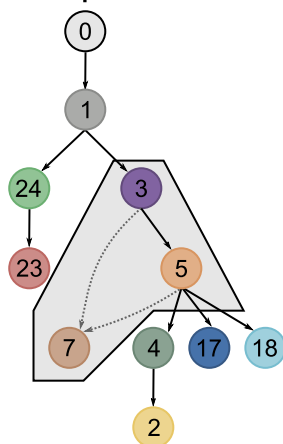

**A34**  
raw

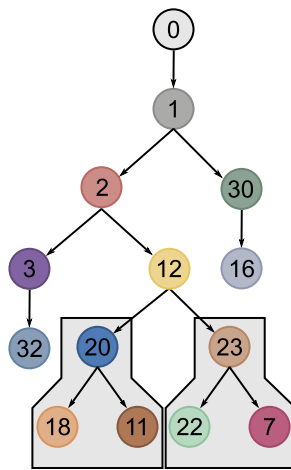

Supplement: S23 Fig — The grey boxes show the parts of the partial clone trees that differ in Gundem et al., where for patient A22, the dark brown subclone with ID 23 is a child of the grey subclone with ID 2, for patient A24, the light blue subclone with ID 13 is a child of the orange subclone with ID 9, for patient A29, there is no uncertainty, the grey subclone with ID 5 is the parent of the dark pink subclone with ID 4 and the light blue subclone with ID 2, subclone 4 is the parent of the gold subclone with ID 3, and subclone 2 is the parent of the dark green subclone with ID 1, for patient A31, the light brown subclone with ID 7 is the child of the dark purple subclone with ID 3, and for patient A34, the darkbrown subclone with ID 11 could either be a child of the orange subclone with ID 18 or of the blue subclone with ID 20, and the pink subclone with ID 7 is a child of the light green subclone with ID 22. Note that the partial clone tree for patient A22 based on the raw CCFs does not contain uncertainty for subclone 8, other than in the tree reported by Gundem et al. Also note that the partial clone tree for patient A29 based on the raw CCFs does not allow the light blue subclone with ID 2 to be a parent of the dark green subclone with ID 1. Colors and subclonal IDs are taken from Gundem et al. (PDF) [file pcbi.1008400.s023.pdf]

A10

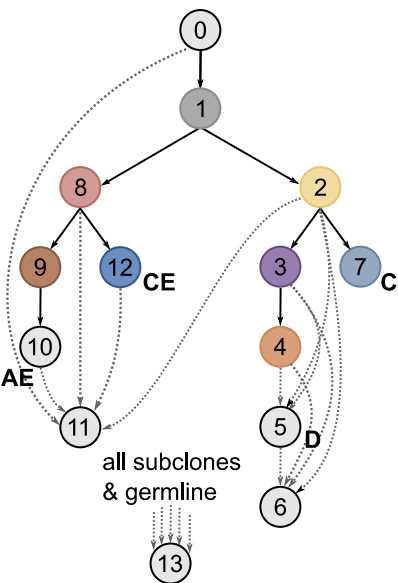

A12

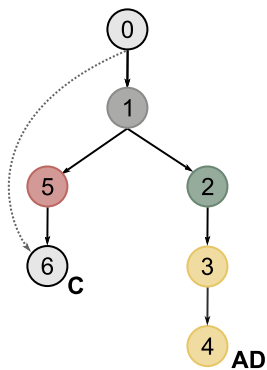

A17

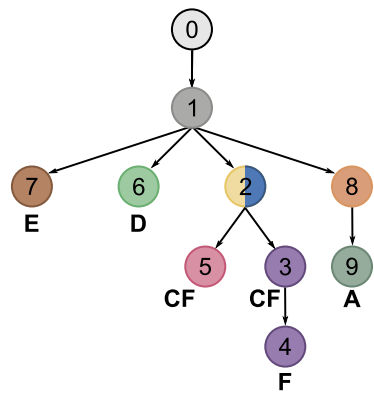

A24

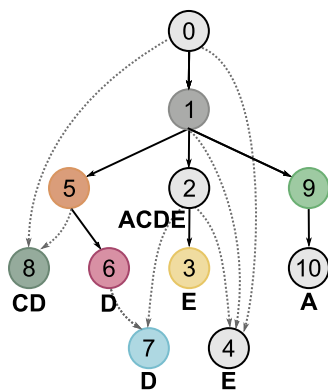

A34

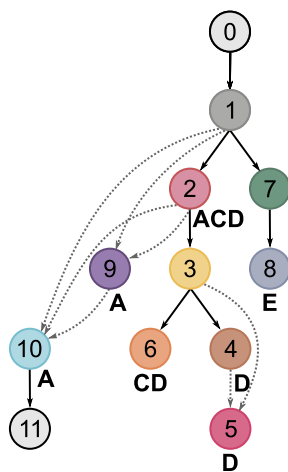

Supplement: S24 Fig — For patient A10, subclone 13 is a possible child of all other subclones and the germline. Subclonal IDs are taken from the PhyloWGS trees. The colors are taken from Gundem et al. and show the mapping of PhyloWGS’ subclones to the ones in Gundem et al. For patient A12 and A17, we mapped multiple PhyloWGS’ subclones to the same subclone of Gundem et al. and in patient A17, we mapped two Gundem et al. subclones to subclone 2 of PhyloWGS. Subclones with a black stroke and grey filling could not be mapped to any Gundem et al. subclone. The letters below and next to the subclones show in which samples the subclones have a frequency higher than or equal to 0.1; ancestral subclones without an explicit labling combine the labeling of their descendants. (PDF) [file pcbi.1008400.s024.pdf]
