# Supplementary material for: Reconstructing tumor evolutionary histories and clone trees in polynomial-time with SubMARine
Source: PLoS Comput Biol. 2021 Jan 19;17(1):e1008400. doi: 10.1371/journal.pcbi.1008400 (PMC7845980; doi:10.1371/journal.pcbi.1008400)
Supplement: S6 Fig — A subclone has uncertain parentage when it has multiple possible parents in the possible parent matrix τ. Line shows mean and gray area standard deviation. (PDF) [file pcbi.1008400.s006.pdf]

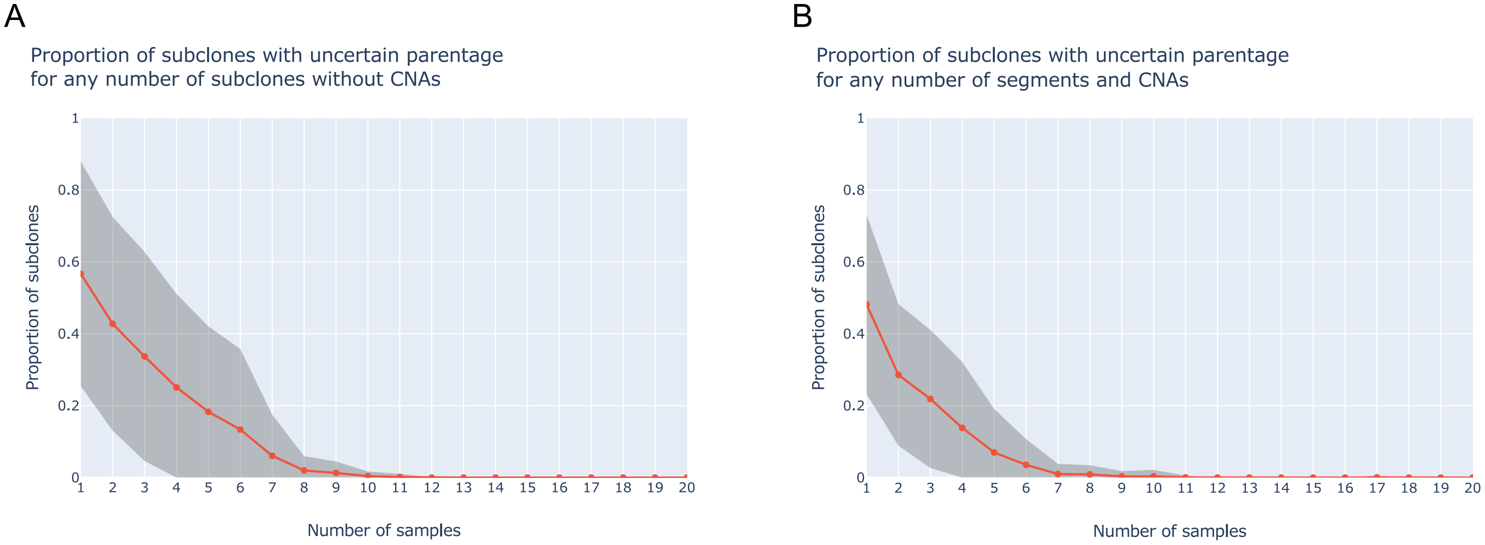

A
Proportion of subclones with uncertain parentage
for any number of subclones without CNAs
Proportion of subclones
Number of samples
0
0.2
0.4
0.6
0.8
1
1 2 3 4 5 6 7 8 9 10 11 12 13 14 15 16 17 18 19 20
B
Proportion of subclones with uncertain parentage
for any number of segments and CNAs
Proportion of subclones
Number of samples
0
0.2
0.4
0.6
0.8
1
1 2 3 4 5 6 7 8 9 10 11 12 13 14 15 16 17 18 19 20
