# Supplementary material for: Reconstructing tumor evolutionary histories and clone trees in polynomial-time with SubMARine
Source: PLoS Comput Biol. 2021 Jan 19;17(1):e1008400. doi: 10.1371/journal.pcbi.1008400 (PMC7845980; doi:10.1371/journal.pcbi.1008400)
Supplement: S8 Fig — (A)–(C) 10 segments, (D)–(F) 20 segments, (G)–(I) 40 segments, (A), (D), (G) 10 CNAs, (B), (E), (H) 20 CNAs, (C), (F), (I) 40 CNAs. A subclone has uncertain parentage when it has multiple possible parents in the possible parent matrix τ. Line shows mean and gray area standard deviation. (PDF) [file pcbi.1008400.s008.pdf]

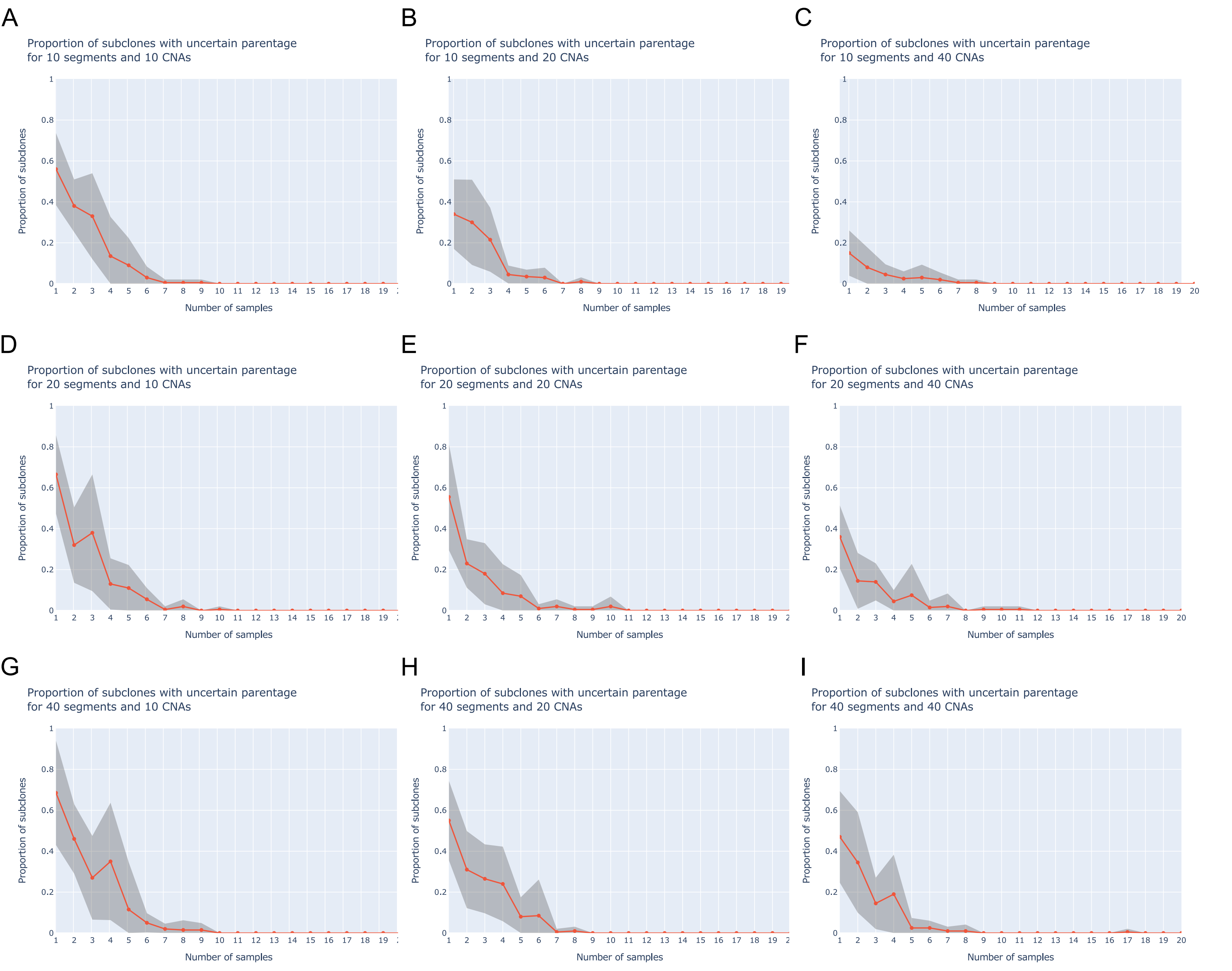
A
Proportion of subclones with uncertain parentage
for 10 segments and 10 CNAs
Proportion of subclones
Number of samples
B
Proportion of subclones with uncertain parentage
for 10 segments and 20 CNAs
Proportion of subclones
Number of samples
C
Proportion of subclones with uncertain parentage
for 10 segments and 40 CNAs
Proportion of subclones
Number of samples
D
Proportion of subclones with uncertain parentage
for 20 segments and 10 CNAs
Proportion of subclones
Number of samples
E
Proportion of subclones with uncertain parentage
for 20 segments and 20 CNAs
Proportion of subclones
Number of samples
F
Proportion of subclones with uncertain parentage
for 20 segments and 40 CNAs
Proportion of subclones
Number of samples
G
Proportion of subclones with uncertain parentage
for 40 segments and 10 CNAs
Proportion of subclones
Number of samples
H
Proportion of subclones with uncertain parentage
for 40 segments and 20 CNAs
Proportion of subclones
Number of samples
I
Proportion of subclones with uncertain parentage
for 40 segments and 40 CNAs
Proportion of subclones
Number of samples
