# Supplementary material for: Reconstructing tumor evolutionary histories and clone trees in polynomial-time with SubMARine
Source: PLoS Comput Biol. 2021 Jan 19;17(1):e1008400. doi: 10.1371/journal.pcbi.1008400 (PMC7845980; doi:10.1371/journal.pcbi.1008400)
Supplement: S12 Fig — Values for subMARs that could be built without a noise buffer are included as 0. (PDF) [file pcbi.1008400.s012.pdf]

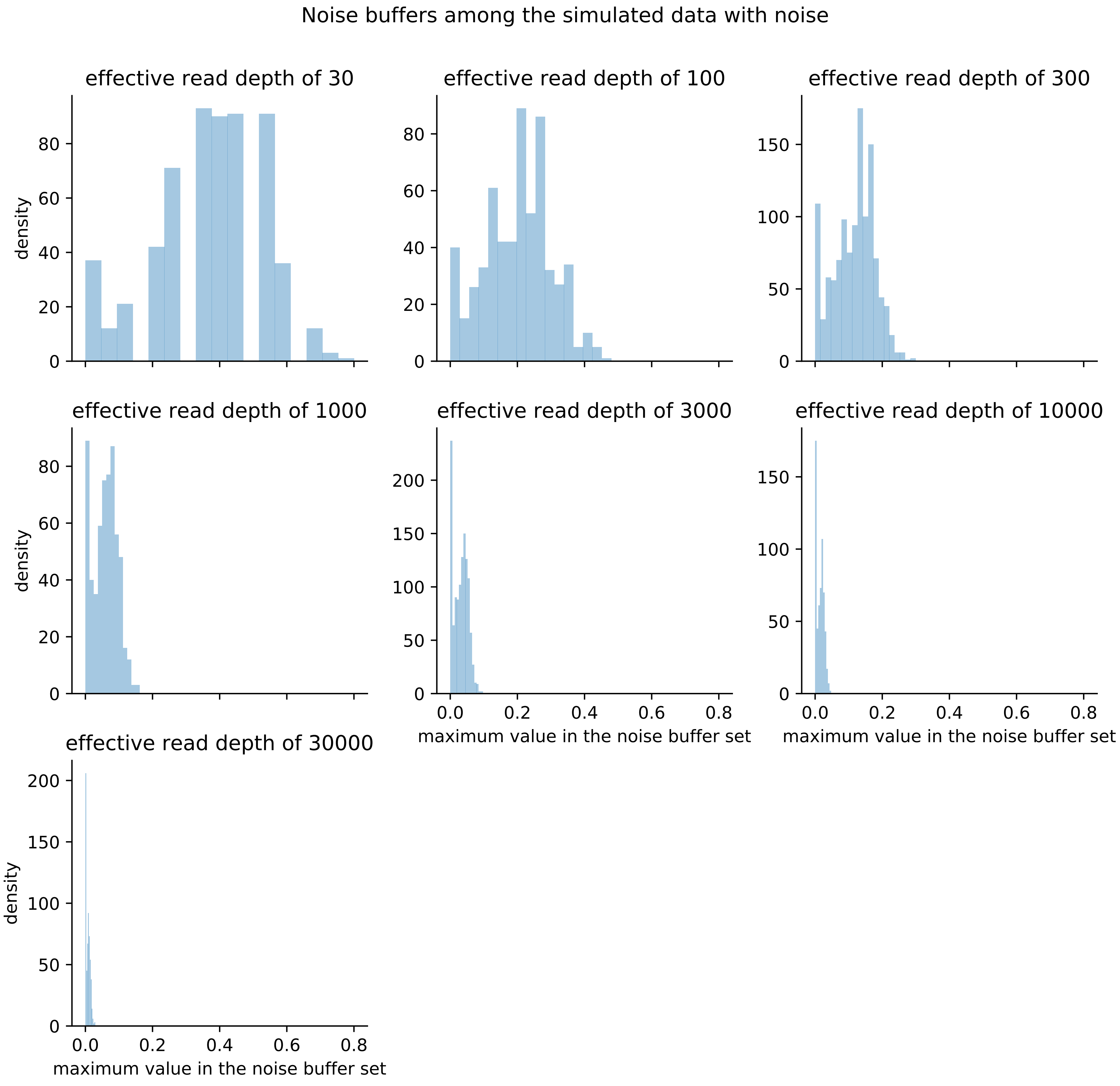
Noise buffers among the simulated data with noise
effective read depth of 30
effective read depth of 100
effective read depth of 300
effective read depth of 1000
effective read depth of 3000
effective read depth of 10000
effective read depth of 30000
density
maximum value in the noise buffer set
0.0
0.2
0.4
0.6
0.8
