# Supplementary material for: Reconstructing tumor evolutionary histories and clone trees in polynomial-time with SubMARine
Source: PLoS Comput Biol. 2021 Jan 19;17(1):e1008400. doi: 10.1371/journal.pcbi.1008400 (PMC7845980; doi:10.1371/journal.pcbi.1008400)
Supplement: S18 Fig — Orange thick bars show the mean recall and percentage of error of the noise-containing data, horizontal orange lines in (A), (C), (E) show the mean recall of the noise-free data. Note that the recall of the noise-free data was calculated by considering only the entries of the upper right triangle of the ancestry matrix Z, while for the noise-containing datasets also the entries of the lower left triangle were considered. (PDF) [file pcbi.1008400.s018.pdf]

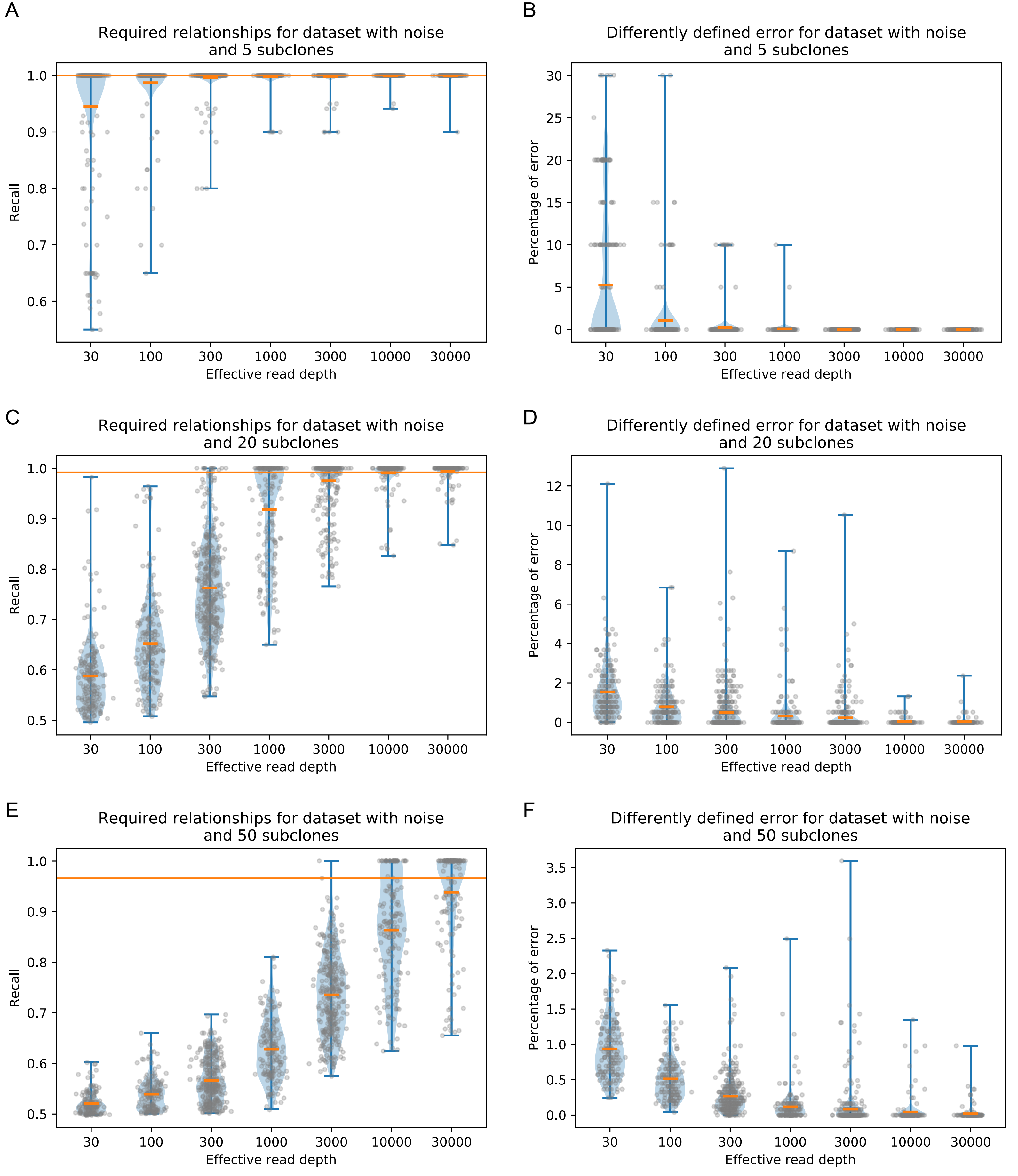

A
Required relationships for dataset with noise and 5 subclones
Recall
Effective read depth
30
100
300
1000
3000
10000
30000
1.0
0.9
0.8
0.7
0.6
B
Differently defined error for dataset with noise and 5 subclones
Percentage of error
Effective read depth
30
25
20
15
10
5
0
C
Required relationships for dataset with noise and 20 subclones
Recall
Effective read depth
0.5
D
Differently defined error for dataset with noise and 20 subclones
Percentage of error
Effective read depth
12
8
6
4
2
E
Required relationships for dataset with noise and 50 subclones
Recall
Effective read depth
F
Differently defined error for dataset with noise and 50 subclones
Percentage of error
Effective read depth
3.5
3.0
2.5
2.0
1.5
0.5
0.0
