# Supplementary material for: Reconstructing tumor evolutionary histories and clone trees in polynomial-time with SubMARine
Source: PLoS Comput Biol. 2021 Jan 19;17(1):e1008400. doi: 10.1371/journal.pcbi.1008400 (PMC7845980; doi:10.1371/journal.pcbi.1008400)
Supplement: S1 Table — (PDF) [file pcbi.1008400.s025.pdf]

**inference rule**	**functional description**	**impact**	**application**
lost allele rules			
i)	$Z_{t+1} \leftarrow f_{lost_{z0}}(\Delta C_A, \Delta C_B, \lambda_{\mathsf{s}}, \lambda_{\mathsf{c}}, \sigma_{\mathsf{s}}, \sigma_{\mathsf{c}}, \pi_{\mathsf{c}}, \pi_{\mathsf{s}_t}, Z_t)$	$Z(k, k') = 0$ (see Eqs (4), (6), and (7) in Section I in S1 Text)	once, and then every time a new 1 is set in Z and an SSM got phased
ii)	$\pi_{\mathsf{s}_0} \leftarrow f_{lost_{pha}}(\Delta C_A, \Delta C_B, \lambda_{\mathsf{s}}, \lambda_{\mathsf{c}}, \sigma_{\mathsf{s}}, \sigma_{\mathsf{c}}, \pi_{\mathsf{c}}, Z_t)$	$\pi_{\mathsf{s}}(j) = \rho(\pi_{\mathsf{c}}(l))$ (see Eq (8) in Section I in S1 Text)	once, and then every time a new 1 is set in Z
equivalence rules			
i)	$Z_0 \leftarrow f_{eq_{z1}}(\mathcal{M}, \lambda_{\mathsf{s}}, \lambda_{\mathsf{c}})$	$Z(k, k') = 1$ (see Eq (13) in Section IV.3 in S1 Text)	once
ii)	$\pi_{\mathsf{s}_0} \leftarrow f_{eq_{samepha}}(\mathcal{M}, \lambda_{\mathsf{s}}, \lambda_{\mathsf{c}}, \pi_{\mathsf{c}})$	$\pi_{\mathsf{s}}(j) = \pi_{\mathsf{c}}(l)$ (see Eq (14) in Section IV.3 in S1 Text)	once
iii)	$\pi_{\mathsf{s}_0} \leftarrow f_{eq_{difpha}}(\mathcal{M}, \lambda_{\mathsf{s}}, \lambda_{\mathsf{c}}, \pi_{\mathsf{c}}, Z_t)$	$\pi_{\mathsf{s}}(j) = \rho(\pi_{\mathsf{c}}(l))$ (see Eq (15) in Section IV.3 in S1 Text)	once, and then every time a new 1 is set in Z
iv)	$Z_{t+1} \leftarrow f_{eq_{z0}}(\mathcal{M}, \Delta C_A, \Delta C_B, \lambda_{\mathsf{s}}, \lambda_{\mathsf{c}}, \sigma_{\mathsf{s}}, \sigma_{\mathsf{c}}, \pi_{\mathsf{c}}, \pi_{\mathsf{s}_t}, Z_t)$	$Z(k, k') = 0$ (see Eqs (16), (17), and (18) in Section IV.3 in S1 Text)	once, and then every time a new 1 is set in Z and an SSM got phased
