# Supplementary material for: Reconstructing tumor evolutionary histories and clone trees in polynomial-time with SubMARine
Source: PLoS Comput Biol. 2021 Jan 19;17(1):e1008400. doi: 10.1371/journal.pcbi.1008400 (PMC7845980; doi:10.1371/journal.pcbi.1008400)
Supplement: S1 Algorithm — (PDF) [file pcbi.1008400.s029.pdf]

S1 Algorithm Functional description of the SubMARine algorithm in extended mode.

Input: $\phi \in \mathbb{R}^{K \times N}, \mathcal{M} \in \{0,1\}^{J \times L}, \Delta C_A \in \mathbb{Z}^{I \times K}, \Delta C_B \in \mathbb{Z}^{I \times K}, \lambda_\mathsf{s} \in \{1, 2, \ldots, K-1\}^J,$
$\lambda_\mathsf{c} \in \{1, 2, \ldots, K-1\}^L, \sigma_\mathsf{s} \in \{0, 1, \ldots, I-1\}^J, \sigma_\mathsf{c} \in \{0, 1, \ldots, I-1\}^L, \pi_\mathsf{c} \in \{A, B\}^L$

Output: ancestry matrix Z, SSM phasing vector π_s, possible parent matrix τ

$\triangleright$ *set 1's through germline rule and 0's through trivial relationships of generalized sum rule*

$Z_0, \pi_{\mathsf{s}_0} \leftarrow \text{initializeCloneTree}(K, J)$

$\triangleright$ *set 1's through equivalence rule based on Eq (13) (Section IV.3 in S1 Text), propagate 1's through partial tree rule (Eq (1) in main text), set SSM phasing through equivalence rules based on Eqs (14) and (15) (Section IV.3 in S1 Text) and lost allele rule based on Eq (8) (Section I in S1 Text)*

$Z_1, \pi_{\mathsf{s}_1} \leftarrow \text{updateOnesAndPhasing}(\mathcal{M}, \Delta C_A, \Delta C_B, \lambda_\mathsf{s}, \lambda_\mathsf{c}, \sigma_\mathsf{s}, \sigma_\mathsf{c}, \pi_\mathsf{c}, \pi_{\mathsf{s}_0}, Z_0)$

$\triangleright$ *set 0's through equivalence and lost allele rules based on Eqs (16), (17), (18), (4), (6), and (7)(Sections IV.3 and I in S1 Text), through crossing rule (Eq (9) in Section III.1 in S1 Text) of generalized sum rule, propagate 0's through partial tree rule*

$Z_2 \leftarrow \text{updateZeros}(\phi, \mathcal{M}, \Delta C_A, \Delta C_B, \lambda_\mathsf{s}, \lambda_\mathsf{c}, \sigma_\mathsf{s}, \sigma_\mathsf{c}, \pi_\mathsf{c}, \pi_{\mathsf{s}_1}, Z_1)$

$\triangleright$ *set 1's and 0's through generalized sum rule with Subpoplar algorithm*

$Z_3, \pi_{\mathsf{s}_3}, \tau_3 \leftarrow \text{useSubpoplar}(K, \phi, \mathcal{M}, \Delta C_A, \Delta C_B, \lambda_\mathsf{s}, \lambda_\mathsf{c}, \sigma_\mathsf{s}, \sigma_\mathsf{c}, \pi_\mathsf{c}, \pi_{\mathsf{s}_1}, Z_2)$

return $Z_3, \pi_{\mathsf{s}_3}, \tau_3$

initializeCloneTree(K, J):

$Z \leftarrow \{-1\}^{K \times K} \cup f_{germ}(K) \cup f_{sum_{triv}}(K)$

$\pi_\mathsf{s} \leftarrow \{-1\}^J$

return Z, π_s

updateOnesAndPhasing$(\mathcal{M}, \Delta C_A, \Delta C_B, \lambda_\mathsf{s}, \lambda_\mathsf{c}, \sigma_\mathsf{s}, \sigma_\mathsf{c}, \pi_\mathsf{c}, \pi_\mathsf{s}, Z)$:

$Z \leftarrow Z \cup f_{eq_{z1}}(\mathcal{M}, \lambda_\mathsf{s}, \lambda_\mathsf{c})$

$Z \leftarrow f_{ptree}(Z)$

$\pi_\mathsf{s} \leftarrow \pi_\mathsf{s} \cup f_{eq_{samepha}}(\mathcal{M}, \lambda_\mathsf{s}, \lambda_\mathsf{c}, \pi_\mathsf{c}) \cup f_{eq_{difpha}}(\mathcal{M}, \lambda_\mathsf{s}, \lambda_\mathsf{c}, \pi_\mathsf{c}, Z) \cup f_{lost_{pha}}(\Delta C_A, \Delta C_B, \lambda_\mathsf{s}, \lambda_\mathsf{c}, \sigma_\mathsf{s}, \sigma_\mathsf{c}, \pi_\mathsf{c}, Z)$

return Z, π_s

updateZeros$(\phi, \mathcal{M}, \Delta C_A, \Delta C_B, \lambda_\mathsf{s}, \lambda_\mathsf{c}, \sigma_\mathsf{s}, \sigma_\mathsf{c}, \pi_\mathsf{c}, \pi_\mathsf{s}, Z)$:

$Z \leftarrow f_{eq_{z0}}(\mathcal{M}, \Delta C_A, \Delta C_B, \lambda_\mathsf{s}, \lambda_\mathsf{c}, \sigma_\mathsf{s}, \sigma_\mathsf{c}, \pi_\mathsf{c}, \pi_\mathsf{s}, Z) \cup f_{lost_{z0}}(\Delta C_A, \Delta C_B, \lambda_\mathsf{s}, \lambda_\mathsf{c}, \sigma_\mathsf{s}, \sigma_\mathsf{c}, \pi_\mathsf{c}, \pi_\mathsf{s}, Z) \cup f_{sum_{cr}}(\phi)$

$Z \leftarrow f_{ptree}(Z)$

return Z

useSubpoplar$(K, \phi, \mathcal{M}, \Delta C_A, \Delta C_B, \lambda_\mathsf{s}, \lambda_\mathsf{c}, \sigma_\mathsf{s}, \sigma_\mathsf{c}, \pi_\mathsf{c}, \pi_\mathsf{s}, Z)$:

initialize δ, ψ, τ

while Z did not converge **do**

  $Z', \delta, \psi, \tau \leftarrow f_{sum_{subp}}(K, \phi, Z, \delta, \psi, \tau)$

  if Z' contains more 1's than Z **then**

    $Z, \pi_\mathsf{s} \leftarrow \text{updateOnesAndPhasing}(\mathcal{M}, \Delta C_A, \Delta C_B, \lambda_\mathsf{s}, \lambda_\mathsf{c}, \pi_\mathsf{c}, \pi_\mathsf{s}, Z')$

    $Z, \pi_\mathsf{s} \leftarrow \text{updateZeros}(\phi, \mathcal{M}, \Delta C_A, \Delta C_B, \lambda_\mathsf{s}, \lambda_\mathsf{c}, \sigma_\mathsf{s}, \sigma_\mathsf{c}, \pi_\mathsf{c}, \pi_\mathsf{s}, Z)$

  else if Z' contains more 0's than Z **then**

    $Z \leftarrow f_{ptree}(Z')$

return Z, π_s, τ
