# Supplementary material for: Reconstructing tumor evolutionary histories and clone trees in polynomial-time with SubMARine
Source: PLoS Comput Biol. 2021 Jan 19;17(1):e1008400. doi: 10.1371/journal.pcbi.1008400 (PMC7845980; doi:10.1371/journal.pcbi.1008400)
Supplement: S1 Text — (PDF) [file pcbi.1008400.s030.pdf]

S1 Text. Supporting information.

Reconstructing tumor evolutionary histories and clone trees in polynomial-time with SubMARine

Linda K. Sundermann[1,2,3], Jeff Wintersinger[1,2,3,4], Gunnar Rätsch[5,6], Jens Stoye[7], Quaid Morris[1,2,3,4,8*]

1 Donnelly Centre for Cellular and Biomolecular Research, University of Toronto, Toronto, Ontario, Canada
2 Vector Institute for Artificial Intelligence, Toronto, Ontario, Canada
3 Ontario Institute for Cancer Research, Toronto, Ontario, Canada
4 Department of Computer Science, University of Toronto, Toronto, Ontario, Canada
5 Department of Computer Science, ETH Zurich, Zurich, Zurich, Switzerland
6 Biomedical Informatics, University Hospital Zurich, Zurich, Zurich, Switzerland
7 Faculty of Technology and Center for Biotechnology (CeBiTec), Bielefeld University, Bielefeld, North Rhine-Westphalia, Germany
8 Computational and Systems Biology, Memorial Sloan Kettering Cancer Center, New York City, New York, United States of America

* MorrisQ@mskcc.org

Contents

I Details on the lost allele constraint **2**

II Details on partial clone trees **3**
II.1 Ancestry graphs and partial clone trees . . . 3
II.2 Details on the partial tree constraint . . . 4
II.3 Lost allele constraint for partial clone trees . . . 4
II.4 Valid MAR per construction . . . 6

III Details on SubMARine **6**
III.1 SubMARine in basic mode . . . 6
III.2 Subpoplar, the sum rule algorithm . . . 8
III.3 Extending SubMARine to deal with imprecise subclonal frequencies . . . 14
III.4 SubMARine with SSMs and clonal CNAs . . . 15
III.5 Upper bound on the size of MAR- and subMAR-completing clone trees . . . 16

IV Details on extended SubMARine **16**
IV.1 Computation of implied copy numbers and VAFs . . . 16
IV.2 Same data fit despite different impact matrices . . . 17
IV.3 Equivalence constraints . . . 17
IV.4 Monotonicity restriction . . . 20
IV.5 Example of SubMARine in extended mode . . . 20
IV.6 SubMARine in extended mode . . . 22

V Details on results **27**
V.1 Simulating noise-free subclonal reconstructions 27
V.2 Estimating subclonal frequencies from read counts 28
V.3 Computing the recall and percentage of false positives on the noisy dataset 28
V.4 Preprocessing of TRACERx data 29
V.5 Using SubMARine with raw and adapted Gundem et al. CCFs 29
V.6 Using SubMARine in extended mode on Gundem et al. data 29
V.6.1 Cleaning data 29
V.6.2 Parsing data for PhyloWGS 30
V.6.3 Running PhyloWGS and choosing a clone tree 30
V.6.4 Computing a mapping between subclones of PhyloWGS and Gundem et al. . . 31
V.6.5 Parsing data for SubMARine 31

I Details on the lost allele constraint

The lost allele constraint ensures that no mutation, SSM as well as CNA, gets assigned to an allele already deleted completely. In order to formulate this constraint, we need to define for each of the L CNAs the segment on which it occurs, and the subclone and parental allele it is assigned to. For these features, we use the vectors $\sigma_{\mathsf{c}} \in \{0, 1, \ldots, I-1\}^L$, $\lambda_{\mathsf{c}} \in \{1, 2, \ldots, K-1\}^L$ and $\pi_{\mathsf{c}} \in \{A, B\}^L$, respectively, where I is the number of segments, K is the number of subclones including the germline, and A and B are the two parental alleles. We call the alleles simply A and B because often it is not possible to determine which alleles are maternal or paternal. Note that alleles across segment boundaries are not necessarily the same; thus the A alleles of two segments do not have to be inherited both from either mother or father but one can come from mother and one from father. This is because mutations are phased only locally within one segment and not globally across all segments.

We represent CNAs as *relative* copy numbers, thus as copy number changes, and not as absolute copy numbers. Advantages of this representation are described in [1]. We store the direction and magnitude of the copy number changes for each allele in each segment i and subclone k in the matrices ΔC_A and $\Delta C_B \in \mathbb{Z}^{I \times K}$ as follows:

$$\Delta C_A(i,k) = \begin{cases} -1 & \text{if a copy number loss is assigned to allele } A, \\ 0 & \text{if no copy number change is assigned to allele } A, \\ x \geq 1 & \text{if a copy number gain of } x \text{ copies is assigned to allele } A. \end{cases}$$

The matrix ΔC_B is defined analogously for allele B. The normal copy number of an allele that is not influenced by copy number changes is 1.

For all J SSMs, the segment, subclonal and parental allele assignments are stored in the vectors $\sigma_{\mathsf{s}} \in \{0, 1, \ldots, I-1\}^J$, $\lambda_{\mathsf{s}} \in \{-1, 1, 2, \ldots, K-1\}^J$ and $\pi_{\mathsf{s}} \in \{A, B, -1\}^J$, respectively. A negative value in the vectors λ_{s} and π_{s} indicates that the entry is undefined and the SSM is not assigned to a subclone or allele. We call an SSM *unphased* if it is not assigned to an allele.

Given the mutation assignment information, we can now formally formulate the lost allele constraint with five equations as follows:

1. If both subclones k and k' lose the same allele in the same segment and have no copy of this allele left, they cannot be in an ancestral-descendant relationship:

$$\begin{aligned} Z(k,k') = 0 \text{ if } \exists\ i \in \{0,1,\ldots,I-1\}, \alpha \in \{A,B\} \text{ such that:} \\ \Delta C_\alpha(i,k) = -1 \text{ and } \Delta C_\alpha(i,k') = -1 \\ \text{and} \sum_{k^* \in \mathcal{A}(k)} \Delta C_\alpha(i,k^*) = 0 \text{ and} \sum_{k^* \in \mathcal{A}(k')} \Delta C_\alpha(i,k^*) = 0, \end{aligned} \tag{4}$$

where Z is the ancestry matrix defined in Section "Partial clone trees" in the main text and where the function $\mathcal{A}(k)$ returns all ancestors of subclone k:

$$\mathcal{A}(k) = \{k^* \mid Z(k^*, k) = 1 \text{ for } k^* < k\}.$$

2. If all copies of an allele are lost in a segment of subclone k, its copy number cannot be changed in descendant subclones. Thus, subclone k cannot be the ancestor of subclone k' that contains a copy number change of this allele in the same segment:

$$Z(k, k') = 0 \text{ if } \exists\, i \in \{0, 1, \dots, I-1\}, \alpha \in \{A, B\} \text{ such that:}$$
$$\sum_{k^* \in \mathcal{A}(k)} \Delta C_\alpha(i, k^*) + \Delta C_\alpha(i, k) = -1 \wedge \Delta C_\alpha(i, k') \neq 0. \quad (5)$$

3. If subclone k lost all copies of one allele, it cannot be the ancestor of subclone k' that has at least one SSM that is phased to this allele in the same segment:

$$Z(k, k') = 0 \text{ if } \exists\, i \in \{0, 1, \dots, I-1\}, j \in \{0, 1, \dots, J-1\}, \alpha \in \{A, B\} \text{ such that:}$$
$$\sum_{k^* \in \mathcal{A}(k)} \Delta C_\alpha(i, k^*) + \Delta C_\alpha(i, k) = -1 \text{ and } \lambda_{\mathsf{s}}(j) = k' \text{ and } \sigma_{\mathsf{s}}(j) = i \text{ and } \pi_{\mathsf{s}}(j) = \alpha. \quad (6)$$

4. If subclone k lost all copies of both alleles in one segment, it cannot be the ancestor of subclone k' that has at least one SSM in the same segment:

$$Z(k, k') = 0 \text{ if } \exists\, i \in \{0, 1, \dots, I-1\}, j \in \{0, 1, \dots, J-1\} \text{ such that:}$$
$$\sum_{k^* \in \mathcal{A}(k)} \Delta C_A(i, k^*) + \Delta C_A(i, k) = -1 \text{ and } \sum_{k^* \in \mathcal{A}(k)} \Delta C_B(i, k^*) + \Delta C_B(i, k) = -1$$
$$\text{and } \lambda_{\mathsf{s}}(j) = k' \text{ and } \sigma_{\mathsf{s}}(j) = i. \quad (7)$$

5. If subclone $\lambda_{\mathsf{s}}(j)$ or an ancestral subclone loses all copies of an allele in segment $\sigma_{\mathsf{s}}(j)$, the SSM j needs to be phased to the opposite allele:

$$\pi_{\mathsf{s}}(j) = \rho(\alpha) \text{ if } \exists\, \alpha \in \{A, B\} \text{ such that:}$$
$$\sum_{k^* \in \mathcal{A}(\lambda_{\mathsf{s}}(j))} \Delta C_\alpha(\sigma_{\mathsf{s}}(j), k^*) + \Delta C_\alpha(\sigma_{\mathsf{s}}(j), \lambda_{\mathsf{s}}(j)) = -1. \quad (8)$$

II Details on partial clone trees

II.1 Ancestry graphs and partial clone trees

Ancestry graphs are DAGs where the vertices represent subclones and an edge goes from subclone k to subclone k' if $\phi(k, n) \geq \phi(k', n)$ for all samples $n \in \{0, 1, \dots, N-1\}$ and if k' does not contain any mutation that is already lost in k [2–4]. Every ancestry graph can be represented as a partial clone tree where $Z(k, k') = -1$ if an edge connects k to k', and where $Z(k, k') = 0$ otherwise. To convert a partial clone tree into an ancestry graph, an edge is drawn from subclone k to k' if k is a possible parent of k' (Definition 1). However, not every partial clone tree can be represented as an ancestry graph. It is not possible when a subclone k has a possible parent that has a possible parent k^* that is not an ancestor of k ($Z(k^*, k) = 0$, Fig A.A and A.B). Ancestry graph methods enumerate clone trees as spanning trees. This approach is not intuitive with partial clone trees because not every spanning tree completes the ancestry matrix Z (Fig A.A and A.C).

A

ϕ	0	1
0	1.0	1.0
1	0.4	0.5
2	0.2	0.3
3	0.15	0.15

Z	0	1	2	3
0	0	1	1	1
1	0	0	-1	0
2	0	0	0	-1
3	0	0	0	0

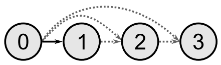

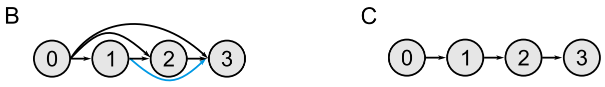

Fig A. Example of (A) a partial clone tree, (B) an ancestry graph and (C) a possible spanning tree. (A) Subclonal frequency matrix ϕ, ancestry matrix Z and corresponding partial clone tree for an example with three subclones. Subclones 2 and 3 have two possible parents but subclone 1 is not an ancestor of subclone 3 ($Z(1,3) = 0$). We assume that no mutations get lost in this example. (B) Only the black edges would be drawn in the ancestry graph when converting the partial clone tree of (A). According to the definition of an ancestry graph however, the blue edge also needs to be present. Hence, the given partial clone tree cannot be transformed into a proper ancestry graph. (C) Spanning tree found in the partial clone tree. However, this spanning tree does not complete the ancestry matrix Z because $Z(1,3) = 0$ and here subclone 1 is an ancestor of 3.

II.2 Details on the partial tree constraint

A tree has the following two properties. First, its ancestral relationships are transitive, meaning that if node k is an ancestor of node k' and node k' is an ancestor of node k'', then node k also has to be an ancestor of node k''. Second, each node except the root, has exactly one parent. Thus, if nodes k and k' are both ancestors of node k'', then either node k has to be an ancestor of node k' or vice versa. Because both properties involve triplets of nodes, which correspond to our subclones, an ancestry matrix describes a tree if both properties are true for all triplets of entries. Below, we combine these two properties in the partial tree constraint.

An entry of the ancestry matrix Z can take three different values, leading to 27 different triplet combinations. Assuming that the subclones are sorted in decreasing order of their average subclonal frequencies, two combinations without undefined values violate the two tree properties and six combinations with undefined values have the potential to violate one of the two tree properties depending on whether the undefined value is set to 1 or 0 (Table A). Observing from enumeration of all possible combinations, all violations have in common that $Z(k', k'') = 1$ and that $Z(k, k')$ and $Z(k, k'')$ have different values. Thus, we can conclude the partial tree constraint:

$$Z(k, k') = Z(k, k'') \text{ if } Z(k', k'') = 1, \text{ for } k < k' < k''.$$

II.3 Lost allele constraint for partial clone trees

The five Eqs (4)–(8), which formulate the lost allele constraint for a complete clone tree, depend on the mutation assignments to segments, parental alleles and subclones, as well as on the definite ancestors of some subclones. Whether there is a *possible* ancestor k° of a subclone k with $Z(k^\circ, k) = -1$ in a partial clone tree does not influence the lost allele constraint because as long as subclone k° is not a definite ancestor of subclone k, its copy number changes have no influence on the allele specific copy numbers of subclone k. Hence, the lost allele constraint does not have to be adapted to be used for a partial clone tree.

Table A. Special ancestral relationship combinations of three subclones.

	ancestral relationships	graphical representation	
1.	$\begin{array}{c\|cc} & k' & k'' \\ \hline k & 0 & 1 \\ k' & & 1 \end{array}$	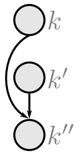	violates single parent property
2.	$\begin{array}{c\|cc} & k' & k'' \\ \hline k & 1 & 0 \\ k' & & 1 \end{array}$	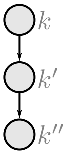	violates transitivity property
3.	$\begin{array}{c\|cc} & k' & k'' \\ \hline k & -1 & 1 \\ k' & & 1 \end{array}$	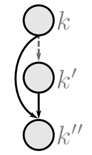	would violate single parent property if $Z(k, k') = 0$
4.	$\begin{array}{c\|cc} & k' & k'' \\ \hline k & 1 & -1 \\ k' & & 1 \end{array}$	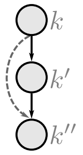	would violate transitivity property if $Z(k, k'') = 0$
5.	$\begin{array}{c\|cc} & k' & k'' \\ \hline k & 1 & 0 \\ k' & & -1 \end{array}$	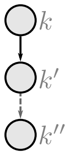	would violate transitivity property if $Z(k', k'') = 1$
6.	$\begin{array}{c\|cc} & k' & k'' \\ \hline k & 0 & 1 \\ k' & & -1 \end{array}$	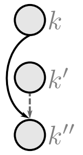	would violate single parent property if $Z(k', k'') = 1$
7.	$\begin{array}{c\|cc} & k' & k'' \\ \hline k & 0 & -1 \\ k' & & 1 \end{array}$	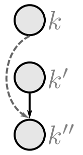	would violate single parent property if $Z(k, k'') = 1$
8.	$\begin{array}{c\|cc} & k' & k'' \\ \hline k & -1 & 0 \\ k' & & 1 \end{array}$	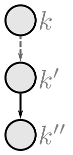	would violate transitivity property if $Z(k, k'') = 1$

Relationships for subclones k, k' and k'', with $0 \leq k < k' < k'' < K$, are shown in an excerpt of the ancestral matrix Z and with a graphical representation. A solid black edge indicates an ancestral-descendant relationship, a gray dashed edge indicates an undefined relationship and no edge between two nodes indicates no ancestral-descendant relationship. The (potentially) violated tree property is indicated for each combination.

II.4 Valid MAR per construction

Given all valid clone trees of a basic clone tree reconstruction problem t, constructing its MAR is trivial. All ancestral relationships that are the same across all clone trees are kept and the ones that differ are set to undefined values. The resulting partial clone tree is always valid. If it was invalid because defined ancestral relationships would violate validity constraints, then this violation would already appear in all clone trees the MAR was constructed from, thus these could not have been valid in the first place. If it was invalid because undefined values would violate the constraints, then in order to satisfy these constraints only one of the defined values would be possible. Hence, all valid clone trees would have to contain this value and consequently, it would not be undefined in the MAR. Therefore, the MAR is valid per construction.

III Details on SubMARine

III.1 SubMARine in basic mode

We now describe in more detail how SubMARine approximates the maximally-constrained ancestral reconstruction problem in basic mode and analyze its runtime. K is the number of subclones including the germline and N is the number of samples.

In basic mode, SubMARine (Algorithm A) takes only the subclonal frequency matrix ϕ for $K-1$ subclones and the germline as input (S4 Fig). These subclones are sorted in decreasing order of their average subclonal frequencies across samples. If two subclones k and k' have the same average subclonal frequencies across all samples, they are sorted according to user-provided IDs, hence the user decides about their order. SubMARine starts by creating an ancestry matrix Z where all relationships are initially undefined ($\mathcal{O}(K^2)$ time, line 3 of Algorithm A). It then performs a small preprocessing phase, propagating the germline rule ($\mathcal{O}(K)$ time, lines 6 and 7), and updating trivial relationships, which are $Z(k,k')=0$ with $k' \leq k$ as a consequence of the generalized sum constraint and the ordering of the subclones ($\mathcal{O}(K^2)$ time, lines 8–10). If user-defined ancestral relationships are given, they are applied, followed by a propagation of the tree rules (lines 12 to 20 in Algorithm A, and Algorithm B). If we do not consider the relationship updates, this is done in $\mathcal{O}(K^2)$ time. Considering propagating the partial tree rule leads to $\mathcal{O}(K^5)$ time. This is because each updated relationship can influence up to K other relationships, which have to be checked. Each of the influenced relationships, can lead to further relationship updates. However, since each of the K^2 relationships is updated at most once, the number of total updates and hence relationship propagations is limited.

Next, the main phase starts, propagating first the crossing rule [5] as another consequence of the generalized sum constraint. This rule states that two subclones k and k' cannot be on the same branch of the clone tree if k has a higher subclonal frequency than k' in sample n but a lower one in sample n'. Because of the order of subclones and the trivial relationships, we know that $Z(k',k)=0$, and hence can implement the crossing rule as:

$$Z(k,k')=0 \text{ if } \phi(k,n)>\phi(k',n) \text{ and } \phi(k,n')<\phi(k',n') \text{ for any } n,n' \in \{0,1,\ldots,N-1\}. \quad (9)$$

Propagating the crossing rule (Eq (9)) naïvley (lines 24–28) without considering relationship updates takes $\mathcal{O}(K^2N^2)$ time. However, because of the ordering of the subclones, we know that the frequency of subclone k, with $k<k'$, is higher than or equal to the frequency of subclone k' in at least one sample. Thus, by checking only whether subclone k has a lower frequency than subclone k' in at least one sample, we can reduce the runtime of the crossing rule to $\mathcal{O}(K^2N)$ time. Its runtime when considering the propagation of the partial tree constraint is $\mathcal{O}(K^5N)$. Afterwards, the generalized sum rule derived from the generalized sum constraint (Eq (3) in main text) is propagated by applying Subpoplar, which also propagates the partial tree rule. In Section III.2, we present this algorithm, which also creates and updates a possible parent matrix τ, indicating the possible parents for each subclone. Furthermore, we show that Subpoplar has a runtime of

Algorithm A Pseudocode of the SubMARine algorithm in basic mode

Input: subclonal frequency matrix ϕ (and user-defined ancestral relationships Z')
Output: ancestry matrix Z, possible parent matrix τ

```
▷ create global variable ancestry matrix Z
K ← |φ|
Z ← {−1}^{K×K}
▷ preprocessing phase
▷ propagate germline rule and update trivial ancestral relationships
for k ← 1, 2, ..., K − 1 do
    Z(0, k) ← 1
for k ← 0, 1, ..., K − 1 do
    for k' ← 0, 1, ..., k do
        Z(k, k') ← 0
▷ apply user-defined constraints on Z if given
if Z' is given then
    for k ← 1, 2, ..., K − 2 do
        for k' ← k + 1, k + 2, ..., K − 1 do
            if Z'(k, k') ≠ −1 then
                if Z(k, k') = −1 then
                    if update_ancestry(Z'(k, k'), k, k') = False then
                        return error message
                else if Z(k, k') ≠ Z'(k, k') then
                    return error message
▷ main phase
▷ propagate crossing rule
N ← |φ(0)|
for k ← 1, 2, ..., K − 2 do
    for k' ← k + 1, k + 2, ..., K − 1 do
        if φ(k, n) > φ(k', n) and φ(k, n') < φ(k', n') for any n, n' ∈ {0, 1, ..., N − 1} then
            if update_ancestry(0, k, k') = False then
                return error message
▷ propagate generalized sum rule, which may lead to further partial tree rule propagations
create global variables needed for Subpoplar algorithm (Section III.2), including possible parent
matrix τ
call Subpoplar algorithm and store returned value in variable x
if x = True then
    return Z, τ
else
    ▷ no valid subMAR exists
    return error message
```

Algorithm B update_ancestry(v, k, k')

Input: value v to which the ancestry matrix Z gets updated, indices k, k' of subclones, whose relationship gets updated, (global variables)

Output: whether $Z(k, k')$ can take the value v

if $Z(k, k') = v$ **then**
    return True
if $Z(k, k') \neq -1$ **then**
    return False
$Z(k, k') \leftarrow v$
for each entry $Z(i, i')$ that should get updated to v' because of partial tree rule propagation of $Z(k, k') = v$ **do**
    if update_ancestry(v', i, i') = **False then**
        return False
return True

$\mathcal{O}(K^3N + K^5)$, which becomes the overall runtime of SubMARine.

At the end, SubMARine returns the subMAR, consisting of the ancestry matrix Z and the subclonal frequency matrix ϕ, as well as the possible parent matrix τ.

It is possible for a user to define relationships for subclones. These relationships are set after the initial ancestry matrix is created and are not allowed to be changed. If a constraint conflicts with one of the user-defined relationships, no subMAR can be found.

III.2 Subpoplar, the sum rule algorithm

Here we describe our generalized sum rule algorithm Subpoplar, which is based on two key constraints: first, in a valid, complete clone tree all subclones must have a single parent, and second, the frequency of a subclone must be greater than or equal to the frequency sum of its children. Furthermore, we analyze Subpoplar's runtime; K is the number of subclones including the germline and N is the number of samples.

Before Subpoplar starts, the possible parent matrix $\tau \in \{0, 1\}^{K \times K}$ is created, following Definition 1 of a possible parent:

$$\tau(k', k) = \begin{cases} 1 & \text{if subclone } k \text{ is a possible parent of subclone } k' \ (k' > k), \\ 0 & \text{otherwise.} \end{cases}$$

In addition, the vector ψ, storing the definite parent for each subclone, is created and initialized with $\psi(k) = -1$ for each subclone k. Also, a frequency matrix $\delta \in \mathbb{R}^{K \times N}$ is created, which indicates the subclonal frequencies that subclones have available to become parents of other subclones without violating the sum constraint. It is initialized with the values of the subclonal frequency matrix ϕ. Creating τ, ψ and δ takes $\mathcal{O}(K^2 + KN)$ time.

Subpoplar processes the subclones in decreasing order of their average frequencies. The version of this algorithm working with subclonal CNAs is shown in Algorithms C to F. For each subclone k, it is checked whether it can be a child of all its possible parents, hence whether its frequency is lower than or equal to the available frequencies of its possible parents in all samples (Algorithm C, lines 1–10). If subclone k has only one possible parent k^*, k is made a definite child (Algorithm C, lines 11–17). This process involves decreasing the available frequency $\delta(k^*, n)$ by $\phi(k, n)$ for each sample n (Algorithm D, lines 1–4). Furthermore, it is checked whether other possible children of subclone k^* can remain its possible children or whether now after updating the available frequency $\delta(k^*, n)$, they would violate the generalized sum rule if they became definite children (Algorithm D, lines 5–22). If they led to a violation, they are removed from the list of possible children. If they were already processed in a previous round of the algorithm and have now without

Algorithm C Pseudocode of the Subpoplar algorithm

Input: global variables K, N, ϕ, Z, λ_{c}, π_{c}, σ_{c}, ΔC_A, ΔC_B, λ_{s}, π_{s}, σ_{s}, and $\mathcal{M}$, possible parent matrix τ, available frequencies δ, definite parents ψ

Output: whether the partial clone tree satisfies the sum constraint

for $k \leftarrow 1, 2, \ldots K-1$ **do**
    for each $k^* \in \{k^* \mid \tau(k, k^*) = 1\}$ **do**
        $\triangleright$ *if k^* cannot be possible parent of k*
        if $\psi(k) \neq k^*$ **and** $\phi(k, n) > \delta(k^*, n)$ **for any** $n \in \{0, \ldots, N-1\}$ **then**
            $\tau(k, k^*) \leftarrow 0$
            $\mathfrak{D} \leftarrow \{k^\circ \mid Z(k^*, k^\circ) \neq 0$ **and** $k^\circ < k\}$
            $\triangleright$ *if no possible descendant of k^* is possible parent of k*
            if $\tau(k, k^\circ) = 0 \ \forall \ k^\circ \in \mathfrak{D}$ **then**
                if update_ancestry_subpoplar(0, k^*, k, k) = **False then**
                    return False
    $\mu \leftarrow \{k^* \mid \tau(k, k^*) = 1\}$
    $\triangleright$ *if k has only one possible parent k^* and is not yet its definite child*
    if $|\mu| = 1$ **and** $\psi(k) = -1$ **then**
        if make_def_child($\mu(0)$, k, k) = **False then**
            return False
    else if $|\mu| < 1$ **then**
        return False
for $k \leftarrow 2, 3, \ldots K-1$ **do**
    $\triangleright$ *if k does not have a definite parent yet*
    if $\psi(k) = -1$ **then**
        $\triangleright$ *ensure that k is descendant of definite ancestors*
        $\mu \leftarrow \{k^* \mid \tau(k, k^*) = 1\}$
        $\mathfrak{l} \leftarrow$ lowest common ancestor of subclones in μ
        if update_ancestry_subpoplar(1, $\mathfrak{l}$, k, $K-1$) = **False then**
            return False
return True

Algorithm D make_def_child(k^*, k, l)

Input: index k^* of parental subclone, index k of child subclone, currently processed subclone l in generalized sum rule algorithm, (global variables)

Output: whether subclone k can become a child of subclone k^*

$\delta(k^*, n) \leftarrow \delta(k^*, n) - \phi(k, n)\ \forall\ n \in \{0, 1, \ldots, N-1\}$
if $\delta(k^*, n) < 0$ **for any** $n \in \{0, 1, \ldots, N-1\}$ **then**
    return False
$\psi(k) \leftarrow k^*$
$\triangleright$ *already processed k' which is possible child of k^* but not its definite child*
$\nu \leftarrow \{k' \mid \tau(k', k^*) = 1$ **and** $k' < l$ **and** $\psi(k') = -1\}$
while $\nu \neq \{\}$ **do**
    $k' \leftarrow \nu$.pop(0)
    $\triangleright$ *if k^* cannot be possible parent of k'*
    if $\phi(k', n) > \delta(k^*, n)$ **for any** $n \in \{0, 1, \ldots, N-1\}$ **then**
        $\tau(k', k^*) \leftarrow 0$
        $\mathfrak{D} \leftarrow \{k^\circ \mid Z(k^*, k^\circ) \neq 0$ **and** $k^\circ < k'\}$
        $\triangleright$ *if no possible descendant of k^* is possible parent of k'*
        if $\tau(k', k^\circ) = 0\ \forall\ k^\circ \in \mathfrak{D}$ **then**
            if update_ancestry_subpoplar(0, k^*, k', l) = **False then**
                return False
        $\mu \leftarrow \{k^\circ \mid \tau(k', k^\circ) = 1\}$
        $\triangleright$ *if k' has only one possible parent k°, which is not yet definite parent*
        if $|\mu| = 1$ **and** $\psi(k') = -1$ **then**
            if make_def_child($\mu(0)$, k', l) = **False then**
                return False
    $\nu \leftarrow \{k'' \mid \tau(k'', k^*) = 1$ **and** $k' < k'' < l$ **and** $\psi(k'') = 0\}$
if update_ancestry_subpoplar(1, k^*, k, l) = **False then**
    return False
return True

k^* only one possible parent left, the complete child updating process is performed recursively. At the end of each such process, the relationship $Z(k, k')$ is updated (Algorithm E). In basic mode of SubMARine, every update of an ancestral relationship also leads to a propagation of the partial tree rule (Eq (1) in main text and Algorithm E, lines 33–35). In extended mode, in addition to the partial tree rule, SSM phases (Algorithm E, lines 20 and 21) and absent relationships (Algorithm E, lines 22 and 23 and Algorithm F) are also propagated to satisfy the equivalence and lost allele constraints. At the end of Subpoplar, if a subclone does not have a definite parent yet, the lowest common ancestor of all its possible parents is made its ancestor to use all information present in the data to eventually propagate further relationships (Algorithm C, lines 18–25).

No matter whether Subpoplar was called from the basic or extended version of SubMARine, without children, relationship and SSM phasing updates, Subpoplar (Algorithm C) needs $\mathcal{O}(K^2N + K^3)$ time because for each subclone and all of its possible parents, the frequencies in all samples have to be compared, and furthermore, all descendants of all possible parents might have to be processed. Making a child the definite child of its definite parent k^* (Algorithm D) without considering relationship updates and recursive calls, takes $\mathcal{O}(KN + K^2)$ time because for each possible child of k^* the frequencies in all samples have to be considered and eventually all possible descendants of k^* have to be checked to be possible ancestors of the possible children. We now start to consider single relationship updates when updating children, differentiating the two possibly required values. If a possible child k' cannot be a descendant of k^*, an absent relationship $(Z(k^*, k') = 0)$ is created with Algorithm E. Without considering further updates, updating to this absent relationship takes $\mathcal{O}(K^2)$ time because possible ancestors of k^* have to be checked to have possible descendants that are possible parents of k', and furthermore, the new relationship $Z(k^*, k')$ can influence up to K relationships through the partial tree rule, which needs to be checked. Hence, making a child k the definite child of its definite parent k^* and considering only absent relationship updates of this action, takes $\mathcal{O}(KN + K^3)$ time, where one of the factors K from updating children is now superseded with K^2. The second relationship update to consider when updating children is a positive relationship update $(Z(k^*, k) = 1)$. In basic mode, without SSM phasing and subclonal CNAs, and without further updates, this does not increase the asymptotic run time of updating children (Algorithm E). However, in extended mode, propagating SSM phasing and absent relationships to satisfy the equivalence and lost allele constraints takes an additional $\mathcal{O}(K^2(IJK + JL^2K)) = \mathcal{O}(K^3JK + JL^2K^2)$ time (Algorithm E, line 20, and Algorithm F, line 3 and equations mentioned therein), where I is the number of segments, J is the number of SSMs and L is the number of CNAs. Without further updates, making a child the definite child of its definite parent thus takes $\mathcal{O}(KN + IJK^4 + JL^2K^2)$, where analogously to the basic mode one factor K gets superseded by the complexity of the relationship update. Now, updating ancestral relationships can propagate further updates, yet, since each relationship is updated at most once, the number of total updates and hence relationship propagations is limited. Because there are only K^2 relationships, the total runtime of the Subpoplar algorithm with all updates in basic mode is $\mathcal{O}(K^3N + K^5)$ and in extended mode is $\mathcal{O}(K^3N + K^6IJ + K^6JL^2)$.

Algorithm E update_ancestry_subpoplar(v, k^*, k, l)

Input: value v to which the ancestry matrix Z gets updated, indices k^*, k of subclones, whose relationship gets updated, currently processed subclone l in generalized sum rule algorithm, (global variables)

Output: whether $Z(k^*, k)$ can take the value v

if $Z(k^*, k) = v$ **then**
  return True
if $Z(k^*, k) \neq -1$ **then**
  return False
$Z(k^*, k) \leftarrow v$
▷ *if ancestor-descendant relationship gets created, the possible parent matrix needs to be updated because multiple parts could change*
if $v = 1$ **then**
  update τ
if $v = 0$ **then**
  $\tau(k, k^*) \leftarrow 0$
▷ *if k was already processed and does not have a definite parent yet*
if $k < l$ **and** $\psi(k) = -1$ **then**
  $\mu \leftarrow \{k^\circ \mid \tau(k, k^\circ = 1)\}$
  ▷ *if k has only one possible parent k°*
  if $|\mu| = 1$ **then**
    if make_def_child($\mu(0)$, k, l) = **False then**
      return False
▷ *if ancestor-descendant relationship gets gets created*
if $v = 1$ **then**
  if propagation of SSM phasing (Eqs (8) and (15)) leads to constraint violations **then**
    return False
  if propagate_absent_relationships(l) = **False then**
    return False
▷ *if ancestral relationship is set absent*
else if $v = 0$ **then**
  $\mathfrak{A} \leftarrow \{k^\circ \mid Z(k^\circ, k^*) \neq 0$ **and** $Z(k^\circ, k) \neq 0\}$
  for $k^\circ \in \mathfrak{A}$ **do**
    $\mathfrak{D} \leftarrow \{k^\bullet \mid Z(k^\circ, k^\bullet) \neq 0$ **and** $k^\bullet < k\}$
    ▷ *if (possible) ancestor k° of k^* has no (possible) descendant $k^\bullet$ that is possible parent of k and is not possible parent itself*
    if $\tau(k, k^\bullet) = 0 \; \forall \; k^\bullet \in \mathfrak{D}$ **and** $\tau(k, k^\circ) = 0$ **then**
      if update_ancestry_subpoplar(0, k°, k, l) = **False then**
        return False
for each entry $Z(i, i')$ that should get updated to v' because of partial tree constraint propagation of $Z(k^*, k) = v$ **do**
  if update_ancestry_subpoplar(v', i, i', l) = **False then**
    return False
return True

Algorithm F propagate_absent_relationships(l)

Input: currently processed subclone l in generalized sum rule algorithm, (global variables)
Output: whether a necessary absent ancestral relationship can be propagated

```
for k ← 1, 2, ..., K − 2 do
    for k′ ← k + 1, k + 2, ..., K − 1 do
        if an absent ancestral relationship needs to be applied for subclones k and k′ for any
        segment i ∈ {0, 1, ..., I − 1} because of Eqs (4), (6), (7), (16), (17) or (18) then
            if update_ancestry_subpoplar(0, k, k′, l) = False then
                return False
return True
```

III.3 Extending SubMARine to deal with imprecise subclonal frequencies

The subclonal frequency matrix ϕ impacts directly the result of the generalized sum rule but not the result of any other inference rule (Tables 1 in main text and S1). The matrix is direct input to the crossing rule and Subpoplar. Indirectly, it influences setting the trivial relationships via the ordering of subclones. Because subclonal frequencies cannot be measured precisely from bulk cancer sequencing data but are inferred from noisy mutational frequencies, it is possible that no valid partial clone tree exists that satisfies the generalized sum constraint even though the infinite site assumption holds.

In order to deal with the issue of imprecise subclonal frequencies, we developed a noise-buffered version of SubMARine. We first describe how a minimum noise buffer uniform across subclones and samples is found in polynomial time and then how a set of subclone- and sample-specific buffers can be found.

In the original version of Subpoplar as described in Section III.2, the algorithm checks for each subclone whether its subclonal frequencies are lower than or equal to the available frequencies of its possible parents. All possible parents for which this is not the case, are discarded. However, if the subclonal frequencies are imprecise, it can happen that a subclone k' cannot be a child of any subclone k, not even of the germline. Hence, the generalized sum rule would require setting all entries $Z(k, k') = 0$ but because $Z(0, k') = 1$ as a consequence of the germline rule, no valid partial clone tree exists. To enable finding a partial clone tree also in these cases, we introduce the use of a noise buffer. This buffer is added to the parental frequencies, and leads to Subpoplar discarding possible parents only if the subclonal frequencies of a possible child are greater than the available frequencies of the possible parents plus this buffer b. This leads to the following changes in Subpoplar where we use the buffer b:

- Algorithm C, line 4: **if** $\psi(k) \neq k^*$ **and** $\phi(k, n) > \delta(k^*, n) + b$ **for any** $n \in \{0, \ldots, N-1\}$
- Algorithm D, line 2: **if** $\delta(k^*, n) + b < 0$ **for any** $n \in \{0, 1, \ldots, N-1\}$
- Algorithm D, line 10: **if** $\phi(k', n) > \delta(k^*, n) + b$ **for any** $n \in \{0, 1, \ldots, N-1\}$

Also, we extend the crossing rule used in SubMARine to use the noise buffer b:

$$Z(k, k') = 0 \text{ if } \phi(k, n) > \phi(k', n) \text{ and } \phi(k, n') + b < \phi(k', n') \text{ for any } n, n' \in \{0, 1, \ldots, N-1\}.$$

Whenever no valid partial clone tree exists for a subclonal frequency matrix ϕ, we use a binary search to identify the minimal uniform noise buffer needed to permit a valid solution. However, while this buffer is required for some subclones, it might lead to other subclones having more possible parents than necessary to find a valid partial clone tree. This leads to a subMAR with more uncertainty than needed and to subMAR-completing trees varying in their data fit (Fig B). To prevent this from happening, SubMARine attempts to find in polynomial time, starting from the subMAR computed with the minimum uniform buffer, the subclone- and sample-specific noise buffer set and its corresponding subMAR, such that all completing clone trees have as little negative frequencies in their available frequency matrix δ as possible. For this purpose, for all subclones having multiple possible parents, the available frequencies of all their possible parents get collected. The best possible subclone- and sample-specific buffer set is the one in which the lowest possible buffer is chosen for each subclone with multiple parents. Note that for all subclones that have only one possible parent, we choose the uniform buffer b as their specific buffer because their buffer has no influence on computing negative available frequencies. If a valid subMAR exists for the best buffer set, SubMARine reports this subMAR and buffer set. Otherwise, SubMARine identifies the second best possible set and if a subMAR exists for it, reports this buffer set and subMAR. This second best set is the one where the subclone with the lowest second possible buffer chooses this buffer, and all other subclones with multiple possible parents choose their lowest

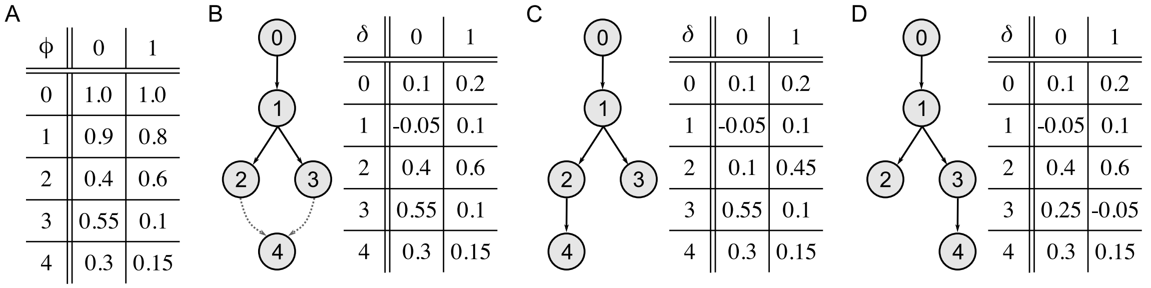

A

ϕ	0	1
0	1.0	1.0
1	0.9	0.8
2	0.4	0.6
3	0.55	0.1
4	0.3	0.15

B

δ	0	1
0	0.1	0.2
1	-0.05	0.1
2	0.4	0.6
3	0.55	0.1
4	0.3	0.15

C

δ	0	1
0	0.1	0.2
1	-0.05	0.1
2	0.1	0.45
3	0.55	0.1
4	0.3	0.15

D

δ	0	1
0	0.1	0.2
1	-0.05	0.1
2	0.4	0.6
3	0.25	-0.05
4	0.3	0.15

Fig B. Example in which a uniform noise buffer leads to subMAR-completing trees with different data fit. (A) Subclonal frequency matrix ϕ for the germline and four subclones across two samples. (B) Valid subMAR and available frequency matrix δ when applying SubMARine with the lowest uniform noise buffer of 0.05 for this dataset. (C) SubMAR-completing tree where subclone 4 is a child of subclone 2. The available frequencies of subclone 2 stay positive. (D) SubMAR-completing tree where subclone 4 is a child of subclone 3. However, in order to become a child, the noise buffer is required. Thus, subclone 3 has a negative available frequency in sample 1. Hence, this tree fits the data worse than the tree in (C).

possible buffer. If no valid subMAR exists for this second best set, SubMARine does not search for the third best one because in order to find it, all buffer combinations have to be considered which cannot be done in polynomial time anymore. Hence, SubMARine informs about the minimum uniform noise buffer and reports it along with the corresponding subMAR.

Instead of comparing all noise buffer combinations to find the best possible subclone- and sample-specific noise buffer set, we offer a different approach. Starting from the reported subMAR and using the uniform noise buffer, SubMARine can apply a depth-first search to find all completing clone trees that have as little negative frequencies in their available frequency matrix δ as possible and constructs their MAR. Whenever a clone tree t with an associated available frequency matrix δ is complete, SubMARine computes the amount of negative available frequency $\delta^{-}_{tree}(\delta)$ of this tree:

$$\delta^{-}_{tree}(\delta) = \sum_{k=0}^{K-1} \sum_{n=0}^{N-1} \min(0, \delta(k, n)).$$

If this frequency is higher than the one of the previous completed tree or trees, they are discarded and the new clone tree t is kept. SubMARine computes the negative available frequency while completing a clone tree, thus can discard a partial clone tree as soon as its frequency gets smaller than the so far best one. When all trees got enumerated, SubMARine builds the MAR for the trees having as little negative available frequencies as possible and reports it along with this frequency and the used subclone- and sample-specific buffer set. Note that instead of keeping enumerated complete clone trees in memory, SubMARine stores in a $K \times K$ matrix which subclonal relationships have been set to present and/or absent in the already enumerated trees. Whenever a tree with a better negative available frequency is found, the relationships in this matrix are set in accordance to this new tree.

III.4 SubMARine with SSMs and clonal CNAs

When in addition to the subclonal frequency matrix ϕ, also SSMs and clonal CNAs are given, SubMARine can be used without any changes to build a subMAR, which describes the set of clone trees of all valid clone trees fitting this setting. The lost allele constraint, which needs to be satisfies when CNAs are given and usually requires knowing the subclone and allele assignment of SSMs, does not have to be propagated. The reason is that as long as only one allele of a segment is deleted by a clonal CNA, SSMs in this segment can be phased to the other allele, which is not affected by

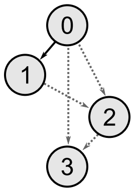

Fig C. Example of a partial clone tree that describes a clone tree not completing it. Subclones 2 and 3 have two possible parents and subclone 1 cannot be an ancestor of subclone 3. When choosing subclone 2 as parent for subclone 3, and subclone 1 as parent for subclone 2, subclone 1 has to be an ancestor of subclone 3 because of the transitivity property of the tree constraint. Then however, this constructed tree does not complete the partial clone tree anymore.

any other CNA. Only if there are clonal losses on both alleles in one segment that also contains SSMs, no valid clone tree exists for this input. We test for this special scenario with a verification step prior to SubMARine that requires the parental allele assignment of the clonal CNAs, and the segment assignment of the CNAs and SSMs. The verification adds the term $\mathcal{O}(L + J)$ to the runtime of SubMARine, where L is the number of CNAs and J is the number of SSMs.

III.5 Upper bound on the size of MAR- and subMAR-completing clone trees

The MAR and the subMAR are a summary of the set of clone trees that complete them and that are all valid. Counting the number of all valid clone trees given a basic clone tree reconstruction problem was shown to be #P-complete [6]. Hence, we derive an upper bound in polynomial time by considering the *possible parents* of each subclone. A possible parent of a subclone includes all ancestral subclones of which this subclone is a child or could become a child. Note that a subclone could also have a single possible parent. Without application of the Subpoplar algorithm (Section III.2), a possible parent is formally defined as follows:

Definition 1 (Possible parent). Subclone k is a possible parent of subclone k' if

$$(Z(k,k') = 1 \text{ or } Z(k,k') = -1) \text{ and } Z(k^\circ,k') \neq 1 \text{ for all } k^\circ \text{ with } k < k^\circ < k'.$$

If the Subpoplar algorithm was applied, possible parents of a subclone k' are indicated in row $\tau(k')$ of the possible parent matrix τ, which is returned by the algorithm.

The number of trees in the summary set of clone trees can easily be computed as follows:

$$\prod_{k=1}^{K-1} \#\text{possible parents of subclone } k. \tag{10}$$

However, because this set can contain clone trees that do not complete the MAR or subMAR (Fig C), its size is an upper bound.

IV Details on extended SubMARine

IV.1 Computation of implied copy numbers and VAFs

The data fit to the experimentally-derived average copy numbers of the segments and the VAFs of the SSMs depends on the average copy numbers and VAFs implied by the clone tree and mutation assignments. Here, we describe how these can be computed and which role the ancestral relationships between subclones play.

The implied average allele-specific copy number for allele A in segment i of sample n can be computed as:

$$\hat{c}_{A_{i,n}} = 1 + \sum_{k} \Delta C_A(i,k) \cdot \phi(k,n), \tag{11}$$

where 1 is the normal copy number of the allele and $\Delta C_A(i,k)$ is the copy number change of allele A in segment i of subclone k as defined in Section I. $\hat{c}_{B_{i,n}}$ can be expressed analogously. Note that $\hat{c}_{A_{i,n}}$ and $\hat{c}_{B_{i,n}}$ do not depend on the ancestral relationships between subclones.

The implied VAF of SSM j in sample n can be computed as:

$$\hat{p}_{j,n} = \frac{\hat{s}_{j,n}}{\hat{c}_{A_{\sigma_{\mathsf{s}}(j),n}} + \hat{c}_{B_{\sigma_{\mathsf{s}}(j),n}}}, \tag{12}$$

where $\hat{s}_{j,n}$ is the mutant copy number of the SSM and is computed as follows:

$$\hat{s}_{j,n} = \phi(\lambda_{\mathsf{s}}(j),n) + \sum_{k' \in \mathcal{D}(\lambda_{\mathsf{s}}(j))} \Delta C_{\pi_{\mathsf{s}}(j)}(\sigma_{\mathsf{s}}(j),k') \cdot \phi(k',n) + \Gamma,$$

where the function $\mathcal{D}(k)$ returns all descendants of subclone k:

$$\mathcal{D}(k) = \{k' \mid Z(k,k') = 1 \text{ for } k < k'\},$$

and Γ is defined as follows:

$$\Gamma = \begin{cases} \phi(\lambda_{\mathsf{s}}(j),n) \cdot \Delta C_{\pi_{\mathsf{s}}(j)}(\sigma_{\mathsf{s}}(j),\lambda_{\mathsf{s}}(j)) & \text{if the mutant copy number of SSM } j \text{ is changed by copy number gain } l \text{ in subclone } \lambda_{\mathsf{s}}(j) \text{ as indicated in the impact matrix } \mathcal{M}, \\ 0 & \text{otherwise,} \end{cases}$$

where $\lambda_{\mathsf{s}}(j)$, $\pi_{\mathsf{s}}(j)$ and $\sigma_{\mathsf{s}}(j)$ are the subclone, parental allele and segment assignments of SSM j as defined in Section I, and $\mathcal{M}$ is the impact matrix as defined in Section "Extended SubMARine: Clone tree reconstruction with subclonal CNAs" in the main text. Because the descendants of subclone $\lambda_{\mathsf{s}}(j)$ have an influence on the computation of the VAF, the ancestral relationships between subclones matters.

IV.2 Same data fit despite different impact matrices

In order for two clone trees c and c' with the same subclonal frequencies and mutation assignments to infer the same data fit although they differ in their impact matrices, one of the following conditions has to hold:

1. two subclones with the same copy number change in the same segment on the same allele have to have exactly the same subclonal frequencies in all samples,
2. two sets of subclones with different subclonal frequencies have to result in exactly the same copy number changes in the same segment on the same allele in all samples.

Fig D shows two examples of these conditions.

IV.3 Equivalence constraints

The segment, parental allele and subclonal assignments of CNAs and SSMs are stored in the vectors σ_{c}, π_{c}, λ_{c} and σ_{s}, π_{s}, λ_{s}, respectively, as was defined in Section I.

If a CNA l changes the mutant copy number of an SSM j and both are assigned to different subclones, then the CNA's subclone has to be descendant of the SSM's one:

$$Z(\lambda_{\mathsf{s}}(j),\lambda_{\mathsf{c}}(l)) = 1 \text{ if } \mathcal{M}(j,l) = 1 \text{ and } \lambda_{\mathsf{s}}(j) \neq \lambda_{\mathsf{c}}(l), \tag{13}$$

A

ϕ	0	1
0	1.0	1.0
1	0.4	0.3
2	0.25	0.2
3	0.25	0.2

B

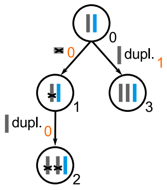

$\mathcal{M}$	0	1
0	1	0

C

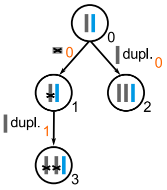

$\mathcal{M}$	0	1
0	0	1

D

ϕ	0	1
0	1.0	1.0
1	0.5	0.4
2	0.4	0.3
3	0.3	0.18
4	0.1	0.12

E

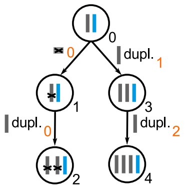

$\mathcal{M}$	0	1	2
0	1	0	0

F

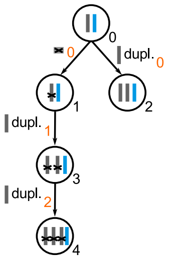

$\mathcal{M}$	0	1	2
0	0	1	1

Fig D. Two examples with different clone trees that have the same subclonal frequencies and mutation assignments but different impact matrices and still infer the same data fit. (A)–(C) Subclones 2 and 3 have the same subclonal frequencies across both samples and contain both a copy number gain. Thus, they influence SSM 0 in the same way which leads to the same VAF. (D)–(F) Whether the mutant copy number of SSM 0 is changed only by copy number change 0 in subclone 2 or by both copy number changes 1 and 2 in subclones 3 and 4 leads to the same VAF (Eq (12)).

Furthermore, the SSM j needs to be phased to the same allele as the CNA l:

$$\pi_{\mathsf{s}}(j) = \pi_{\mathsf{c}}(l) \text{ if } \mathcal{M}(j,l) = 1. \tag{14}$$

If the mutant copy number of an SSM j should not be changed by a CNA l but the CNA is assigned to a descendant subclone in the same segment, the SSM needs to be phased to the opposite allele:

$$\begin{aligned} \pi_{\mathsf{s}}(j) = \rho(\pi_{\mathsf{c}}(l)) \text{ for all } l \in \{0,1,\dots,L-1\} \text{ with} \\ \mathcal{M}(j,l) = 0 \text{ and } Z(\lambda_{\mathsf{s}}(j), \lambda_{\mathsf{c}}(l)) = 1 \text{ and } \sigma_{\mathsf{s}}(j) = \sigma_{\mathsf{c}}(l), \end{aligned} \tag{15}$$

where the function $\rho(\alpha)$ returns the opposite allele:

$$\rho(\alpha) = \begin{cases} A & \text{if } \alpha = B, \\ B & \text{if } \alpha = A. \end{cases}$$

If the phase of an SSM cannot be adapted in order to avoid the unwanted influence of a CNA, the ancestral relationship between the subclone with the SSM and the one with the CNA needs to be absent. There exist three cases where this occurs:

1. If subclone k has an SSM j and subclone k' has a CNA l that are both assigned to the same segment and allele but the CNA should not change the copy number of SSM j, the ancestral relationship between the two subclones has to be absent:

$$\begin{aligned} Z(k,k') = 0 \text{ if } \exists\, l \in \{0,1,\dots,L-1\}, j \in \{0,1,\dots,J-1\} \text{ such that:} \\ \lambda_{\mathsf{s}}(j) = k \text{ and } \lambda_{\mathsf{c}}(l) = k' \text{ and } \sigma_{\mathsf{s}}(j) = \sigma_{\mathsf{c}}(l) \text{ and } \pi_{\mathsf{c}}(l) = \pi_{\mathsf{s}}(j) \text{ and } \mathcal{M}(j,l) = 0. \end{aligned} \tag{16}$$

2. If subclone k has an SSM j, subclone k' has a CNA l in the same segment and either subclone k' or its descendant k'' have another CNAs l' in the same segment on the other allele than l, subclone k cannot be an ancestor of subclone k' if the copy number of SSM j should not be changed by the two CNAs:

$$\begin{aligned} Z(k,k') = 0 \text{ if } \exists\, j \in \{0,1,\dots,J-1\}, l, l' \in \{0,1,\dots,L-1\} \text{ such that:} \\ \lambda_{\mathsf{s}}(j) = k \text{ and } \lambda_{\mathsf{c}}(l) = k' \text{ and } \lambda_{\mathsf{c}}(l') = k' \text{ or } k'' \text{ and } k'' \in \mathcal{D}(k') \\ \text{and } \sigma_{\mathsf{s}}(j) = \sigma_{\mathsf{c}}(l) = \sigma_{\mathsf{c}}(l') \\ \text{and } \pi_{\mathsf{c}}(l) = \rho(\pi_{\mathsf{c}}(l')) \text{ and } \mathcal{M}(j,l) = 0 \text{ and } \mathcal{M}(j,l') = 0, \end{aligned} \tag{17}$$

where the function $\mathcal{D}(k')$ returns all descendants of subclone k' and is defined in Section IV.1.

3. If subclone k' has a CNA l on one allele and is the descendant of a subclone that lost all copies of the other allele in the same segment, it cannot be the descendant of subclone k that has an SSM j in the same segment whose mutant copy number should not be changed by the CNA l:

$$\begin{aligned} Z(k,k') = 0 \text{ if } \exists\, l \in \{0,1,\dots,L-1\}, j \in \{0,1,\dots,J-1\} \text{ such that:} \\ \lambda_{\mathsf{c}}(l) = k' \text{ and } \sum_{k^* \in \mathcal{A}(k')} \Delta C_{\rho(\pi_{\mathsf{c}}(l))}(\sigma_{\mathsf{c}}(l), k^*) = -1 \\ \text{and } \lambda_{\mathsf{s}}(j) = k \text{ and } \sigma_{\mathsf{s}}(j) = \sigma_{\mathsf{c}}(l) \text{ and } \mathcal{M}(j,l) = 0, \end{aligned} \tag{18}$$

where the function $\mathcal{A}(k')$ returns all ancestors of subclone k' and was defined in Section I.

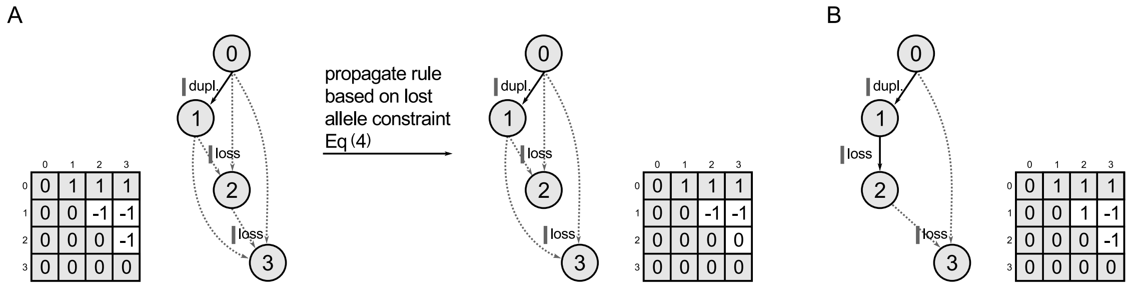

Fig E. Example why extended SubMARine cannot guarantee that defined ancestral relationships in a subMAR have the same value in the corresponding MAR when the monotonicity constraint does not hold. (A) Partial clone tree with ancestry matrix Z before and after inference rule propagation. Subclonal frequencies are not shown because they are not relevant for this example. Gray entries in ancestry matrix Z are trivially a consequence of the ordering of the subclones and the generalized sum rule. Before propagating rules based on the lost allele constraint, subclone 3 can be a descendant of all other subclones. Due to a rule based on Eq (4), subclone 2 is not allowed to be an ancestor of subclone 3 and the corresponding undefined value in the ancestry matrix has to be updated to $Z(2, 3) = 0$. After this update, no more rules affect the undefined values of entry $Z(1, 2)$ or $Z(1, 3)$, hence, SubMARine terminates. (B) If now, the undefined relationship between subclones 1 and 2 was set to a definite one, making subclone 2 a descendant of subclone 1, then because the allele lost in subclone 2 was duplicated in subclone 1 and hence not all copies were lost, subclone 2 is a possible ancestor of subclone 3 again. Thus, there exists a valid and equivalent clone tree that does not complete the subMAR and as a consequence, a defined entry in the subMAR would be undefined in the MAR.

IV.4 Monotonicity restriction

To ensure that the input provided to SubMARine has only copy number changes in one direction per segment and allele, it has to satisfy the following two *monotonicity constraints*:

$$\Delta C_\alpha(i,k) \leq 0 \text{ for all } k \in \{1,2,\ldots,K-1\} \text{ if } \exists\ k^\circ \in \{1,2,\ldots,K-1\}, \alpha \in \{A,B\} \text{ such that: } \Delta C_\alpha(i,k^\circ) < 0,$$

and

$$\Delta C_\alpha(i,k) \geq 0 \text{ for all } k \in \{1,2,\ldots,K-1\} \text{ if } \exists\ k^\circ \in \{1,2,\ldots,K-1\}, \alpha \in \{A,B\} \text{ such that: } \Delta C_\alpha(i,k^\circ) > 0,$$

where ΔC_A and ΔC_B describe the copy number change per allele, segment and subclone and are defined in Section I.

Without the input satisfying the monotonicity constraints, SubMARine could not guarantee that all defined ancestral relationships and SSM phases specified by inference rule propagation in its extended subMAR have the same value in the corresponding extended MAR (Fig E).

IV.5 Example of SubMARine in extended mode

This section contains an example how SubMARine works. For a detailed description with a runtime analysis see Section IV.6.

Given the subclonal frequency matrix ϕ, the impact matrix $\mathcal{M}$, all CNA information, and the SSM assignment to segments and subclones as input (Fig F.C–E), SubMARine in extended mode

A

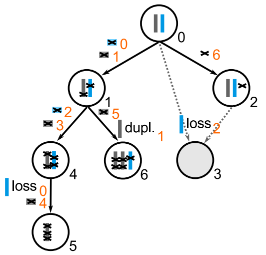

B

Z	0	1	2	3	4	5	6
0	0	1	1	1	1	1	1
1	0	0	**0**	**0**	**1**	**1**	**1**
2	0	0	0	**-1**	**0**	**0**	**0**
3	0	0	0	0	**0**	**0**	**0**
4	0	0	0	0	0	**1**	**0**
5	0	0	0	0	0	0	**0**
6	0	0	0	0	0	0	0

C

ϕ	0	1
0	1.0	1.0
1	0.4	0.3
2	0.25	0.5
3	0.2	0.2
4	0.15	0.17
5	0.1	0.12
6	0.08	0.07

D

$\mathcal{M}$	0	1	2
0	1	0	0
1	0	1	0
2	1	0	0
3	0	0	0
4	0	0	0
5	0	1	0
6	0	0	0

E

$\lambda_c = (5, 7, 3)^T$, $\sigma_c = (0, 0, 0)^T$, $\pi_c = (A, B, A)^T$, $\Delta C_A = (0, 0, 0, 0, 0, 0, 1)$, $\Delta C_B = (0, 0, 0, \text{-}1, 0, \text{-}1, 0, 0)$

$\lambda_s = (1, 1, 4, 4, 5, 6, 2)^T$, $\sigma_s = (0, 0, 0, 0, 0, 0, 0)^T$, $\pi_s = (B, A, B, A, A, A, \text{-}1)^T$

F

$\hat{c}_{A_{0,0}} = 1.08$, $\hat{c}_{B_{0,0}} = 0.7$

$\hat{c}_{A_{0,1}} = 1.07$, $\hat{c}_{B_{0,1}} = 0.68$

G

$\hat{p}_{0,0} = 0.17$, $\hat{p}_{1,0} = 0.27$, $\hat{p}_{2,0} = 0.03$, $\hat{p}_{3,0} = 0.08$, $\hat{p}_{4,0} = 0.06$, $\hat{p}_{5,0} = 0.09$, $\hat{p}_{6,0} = 0.14$

$\hat{p}_{0,1} = 0.1$ $\hat{p}_{1,1} = 0.21$, $\hat{p}_{2,1} = 0.03$, $\hat{p}_{3,1} = 0.1$, $\hat{p}_{4,1} = 0.7$, $\hat{p}_{5,1} = 0.08$, $\hat{p}_{6,1} = 0.29$

Fig F. Valid partial clone tree (A) drawn as partial tree and (B) represented as ancestry matrix Z with (C) subclonal frequency matrix ϕ, (D) impact matrix $\mathcal{M}$ showing influence of CNAs on SSMs, (E) subclone, segment and parental allele assignments for CNAs and SSMs and type of copy number change for CNAs, (F) inferred average copy numbers, and (G) inferred VAFs. This partial clone tree consists of the germline (at the top of (A) with black index 0) and six subclones. We assume that only one segment is given. Allele A is duplicated in subclone 6, allele B gets lost in subclones 3 and 5. Four SSMs are phased to allele A, two are phased to allele B and one is unphased. (Indices of mutations are shown with orange numbers.) Every subclone but subclone 3 has a single possible parent. The two possible parents of subclone 3 are the germline and subclone 2. Thus, the genotype of subclone 3 cannot be unambiguously determined.

builds the valid partial clone tree in Fig F.A in the following order.

Because the monotonicity restriction holds on the CNAs, the preprocessing phase applies the germline rule and sets all trivial relationships $Z(k, k') = 0$ with $k' \leq k$.

Then the main phase starts. First, those of the equivalence and lost allele rules are propagated that lead to 1's in the ancestry matrix Z or that update SSM phasing. Whenever a relationship is updated, the partial tree rule is applied as well. Because CNA 0 of subclone 5 changes the mutant copy numbers of SSMs 0 and 2 of subclones 1 and 4 (Fig F.D and E), the SSMs are phased to the same allele as the CNA (equivalence rule based on Eq (14)) and subclone 5 has to be a descendant of subclones 1 and 4 (equivalence rule based on Eq (13)). In order to satisfy the single parent property of the partial tree constraint (Eq (1) in main text), subclone 1 needs to be an ancestor of subclone 4. CNA 1 of subclone 6 influences the mutant copy numbers of SSMs 1 and 5 of subclones 1 and 6 (Fig F.D and E). Hence, both SSMs are phased to the same allele as the CNA (equivalence based on Eq (14)) and subclone 1 has to be an ancestor of subclone 6 (equivalence rule based on Eq (13)). Because the mutant copy numbers of SSMs 1 and 3 should not be influenced by CNA 0 (Fig F.D), they get phased to the other allele (equivalence rule based on Eq (15)). (SSM 1 was phased to this other allele already because of CNA 1 and Eq (14).) SSM 4 appears after the loss of allele B (Fig F.D and E), hence it has to be phased to allele A (lost allele rule based on Eq (8)).

Second, those of the equivalence and lost allele rules that lead to 0's in Z, and the crossing rule (Eq (9)), which follows from the generalized sum constraint (Eq (3) in main text) and also leads to 0's in Z, are propagated together with the partial tree constraint. Since SSM 4 is phased to the same allele as CNA 1 but its mutant copy number is not influenced by it (Fig F.D), subclone 5 of the SSM cannot be an ancestor of subclone 6 of the CNA (equivalence rule based on Eq (16)). The same reasoning holds for SSM 0 and CNA 2, which is why subclone 1 of the SSM cannot be an ancestor of subclone 3 of the CNA. The transitivity property of the partial tree constraint leads to subclone 3 not able to be an ancestor of subclones 4, 5 and 6. In addition to this, subclone 3 could not be an ancestor of subclone 4 because of the lost allele rule based on Eq (6). The crossing rule forbids subclone 2 to be a descendant of subclone 1 (Fig F.C). Thus, subclone 2 cannot be an ancestor of subclones 4, 5, and 6 (transitivity property of the partial tree constraint).

Third, the generalized sum rule is propagated with Subpoplar. Per default, the germline is the parent of subclone 1. Because subclone 2 cannot be a descendant of subclone 1, the germline is its parent as well. The subclonal frequencies allow subclone 3 to either be a child of the germline or of subclone 2, hence it has two possible parents. Subclones 4, 5 and 6 have only one possible parent left, hence no relationships have to be updated and no inference rule be propagated. Thus, SubMARine terminates and outputs the valid partial clone tree, represented by Z, the SSM phasing vector π_{s} and the possible parent matrix τ (not shown here).

IV.6 SubMARine in extended mode

We now describe in detail the extended mode of SubMARine, which approximates the extended maximally-constrained ancestral reconstruction problem, and analyze its runtime. K is the number of subclones including the germline, N is the number of samples, I is the number of segments, J is the number of SSMs and L is the number of CNAs.

The extended version of SubMARine (Algorithms G–I) takes the subclonal frequencies ϕ, L CNAs with segment, subclonal and phase assignment, σ_{c}, λ_{c} and π_{c}, respectively, the direction and magnitude of copy number changes ΔC_A and ΔC_B for each allele derived from the CNAs, J SSMs with segment and subclonal assignment, σ_{s} and λ_{s}, respectively, and the impact matrix $\mathcal{M}$ of an equivalent clone tree reconstruction problem t as input (Figs S5 and F). (More information on the notation of mutation assignments can be found in Section I.) As in basic mode (Section III.1), the subclones are sorted in decreasing order of their average subclonal frequencies and if two subclones have the same average frequencies, their user-provided IDs decide about their order. Extended SubMARine starts by creating an ancestry matrix Z in which all relationships are initially undefined ($\mathcal{O}(K^2)$ time, Algorithm G, line 3). Additionally, the phases of all SSMs are initialized in the vector π_{s} with the undefined value ($\mathcal{O}(J)$ time, line 7). Then the monotonicity restriction is

Algorithm G Pseudocode of the SubMARine algorithm in extended mode

Input: global variables ϕ, λ_{c}, π_{c}, σ_{c}, ΔC_A, ΔC_B, λ_{s}, σ_{s}, and $\mathcal{M}$
Output: ancestry matrix Z, possible parent matrix τ, SSM phasing vector π_{s}

$\triangleright$ *create global variables*
$K \leftarrow |\phi|$
$Z \leftarrow \{-1\}^{K \times K}$
$J \leftarrow |\lambda_{\mathsf{s}}|$
$L \leftarrow |\lambda_{\mathsf{c}}|$
$I \leftarrow |\Delta C_A(0)|$
$\pi_{\mathsf{s}} \leftarrow \{-1\}^J$
$\triangleright$ *check monotonicity restriction*
if monotonicity restriction (Section IV.4) does not hold **then**
    return error message
$\triangleright$ *preprocessing phase*
$\triangleright$ *propagate germline rule and update trivial ancestral relationships*
for $k \leftarrow 1, 2, \ldots, K-1$ **do**
    $Z(0, k) \leftarrow 1$
for $k \leftarrow 0, 1, \ldots, K-1$ **do**
    for $k' \leftarrow 0, 1, \ldots, k$ **do**
        $Z(k, k') \leftarrow 0$
$\triangleright$ *main phase*
$\triangleright$ *propagate CNA influence on SSMs*
propagate_CNA_influence_on_SSMs()
$\triangleright$ *propagate absent relationships*
propagate_absent_relationships()
$\triangleright$ *propagate crossing rule*
$N \leftarrow |\phi(0)|$
for $k \leftarrow 1, 2, \ldots, K-2$ **do**
    for $k' \leftarrow k+1, k+2, \ldots, K-1$ **do**
        if $\phi(k, n) > \phi(k', n)$ and $\phi(k, n') < \phi(k', n')$ for any $n, n' \in \{0, 1, \ldots, N-1\}$ **then**
            if update_ancestry$(0, k, k')$ = **False then**
                return False
$\triangleright$ *propagate generalized sum rule, which may lead to partial tree rule, absent relationship and SSM phase propagation*
create global variables needed for Subpoplar algorithm (Section III.2), including possible parent matrix τ
call Subpoplar algorithm and store returned value in variable x
if x = **True then**
    return Z, τ
else
    $\triangleright$ *no valid subMAR exists*
    return error message

checked in $\mathcal{O}(K^2 I)$ time (line 9). In the preprocessing phase, the germline rule is introduced ($\mathcal{O}(K)$ time lines 13 and 14), and trivial relationships are set as a consequence of the generalized sum rule and sorting of subclones, i. e., $Z(k, k') = 0$ with $k' \leq k$ ($\mathcal{O}(K^2)$ time, lines 15–17). Then the main phase starts and extended SubMARine takes care that SSMs are influenced by CNAs as indicated by the impact matrix $\mathcal{M}$ (Algorithm G, line 20 and Algorithm H). First, it propagates Eq (13) and phases SSMs to the alleles of CNAs that impact them and creates ancestral-descendant relationships ($Z(k, k') = 1$) between subclones (Algorithm H, lines 1–13). If no other ancestral relationships get propagated by the partial tree rule, which are checked after creating an ancestral-descendant relationship, this takes $\mathcal{O}(JLK)$ time. Because of the possible creation of ancestral-descendant relationships, SSMs not impacted by CNAs that are now in a descendant subclone must be phased to the other allele; this is done by propagating Eq (15) ($\mathcal{O}(JL)$ time, lines 15–21). After ensuring that the equivalence constraints are satisfied so far, the lost allele constraint needs to be checked. This is done by propagating Eq (8) and updating SSM phasing whenever an SSM could be phased to a lost allele otherwise ($\mathcal{O}(JK)$ time, lines 23–29). In total, taking care that SSMs are influenced by CNAs as indicated by the impact matrix $\mathcal{M}$ and that the equivalence and lost allele constraints are satisfied takes $\mathcal{O}(JLK)$ time when the partial tree rule does not lead to the propagation of further relationships. Now, to ensure that the equivalence and lost allele constraints are satisfied, absent ancestral relationships ($Z(k, k') = 0$) are propagated (Algorithm G, line 22 and Algorithm I). First, Eqs (16), (18) and (17) of the equivalence constraint are propagated (Algorithm I, lines 2–17), which takes $\mathcal{O}(JLK^2 + JL^2K)$ time if no other relationships get propagated because of the partial tree rule. Second, Eqs (4), (6) and (7) of the lost allele constraint are propagated (lines 19–37), taking $\mathcal{O}(L^2K + LJK^2)$ time with no relationship updates caused by the partial tree rule. Note that because of the monotonicity restriction, Eq (5) does not have to be considered. In total, propagating absent relationships with Algorithm I takes $\mathcal{O}(JLK^2 + JL^2K)$ time without further updates. Afterwards extended SubMARine uses the crossing rule (Eq (9)), which includes propagating the partial tree rule (Algorithm G, lines 25–29). This can be achieved in $\mathcal{O}(K^3 N)$ time when no other relationships are propagated by the tree rule and the crossing rule is implemented with the trick described in Section III.1. Before considering the last step of extended SubMARine, which is propagating the generalized sum rule with the Subpoplar algorithm, we summarize extended SubMARine's runtime so far. Without relationship updates caused by propagating the partial tree rule, it has a runtime of $\mathcal{O}(K^3N + JLK^2 + JL^2K)$. Because the ancestry matrix has only K^2 ancestral relationships and each relationship is updated at most once, the total runtime of extended SubMARine so far when considering relationship updates of the partial tree rule is simply $\mathcal{O}(K^5N + JLK^4 + JL^2K^3)$. Finally, the generalized sum rule is propagated with the Subpoplar algorithm, which also takes care of the partial tree, the equivalence and the lost allele rules. In Section III.2, we present this algorithm, which also creates and updates a possible parent matrix τ, indicating the possible parents for each subclone. Additionally, we derive its runtime of $\mathcal{O}(K^3N + K^6IJ + K^6JL^2)$, which already considers all possible relationship updates. Hence, the total runtime of extended SubMARine is $\mathcal{O}(K^5N + K^6IJ + K^6JL^2)$.

Extended SubMARine converges when no ancestral relationship or SSM phases can be propagated anymore, which is after the Subpoplar algorithm finishes. Because only undefined relationships and SSM phases are updated and those are finite, it always converges. It returns an extended subMAR as result, which consists of the ancestry matrix Z, the SSM phasing vector π_{s} and the possible parent matrix τ.

It is possible for a user to define relationships for subclones and phases for SSMs. These relationships and phases are set after the initialization of Z and π_{s} (S5 Fig) and are not allowed to be changed. If a constraint conflicts with one of the user-defined relationships, no subMAR can be found.

Like the basic subMAR, the extended subMAR has three important properties for an extended clone tree reconstruction problem t: its defined ancestral relationships and SSM phases are a subset of those in the extended MAR, it is unique, and consequently, all valid and equivalent clone trees of t are completions of the extended subMAR. The reasoning for this follows the same argument as for

the basic subMAR. Only undefined relationships and SSM phases are updated to defined ones and only when, given all other defined values, one of the two possible defined value causes a violation of a validity or equivalence constraint. Because the input data satisfies the monotonicity restriction, no updated value can be transformed back to the undefined value without violating a rule. Hence, the defined values are a subset of those in the extended subMAR. Even though the extended version of SubMARine works with more inference rules, i. e., those belonging to the lost allele and the equivalence constraints, no rule depends on an undefined value in order to update another undefined value. Thus, given a set of initially defined values, the order in which the inference rules are applied does not matter; the extended subMAR is unique.

Algorithm H propagate_CNA_influence_on_SSMs()

Input: (global variables)

Output: whether ancestral relationships and SSM phases can be updated in a way that CNAs influence SSMs as stated in impact matrix $\mathcal{M}$

for $j \leftarrow 0, 1, \ldots, J-1$ **do**
    for $l \leftarrow 0, 1, \ldots, L-1$ **do**
        if $\mathcal{M}(j, l) = 1$ **then**
            ▷ *phase SSM following Eq (14)*
            if $\pi_{\mathsf{s}}(j) = -1$ **then**
                $\pi_{\mathsf{s}}(j) = \pi_{\mathsf{c}}(l)$
            else if $\pi_{\mathsf{s}}(j) \neq \pi_{\mathsf{c}}(l)$ **then**
                return False
            ▷ *update ancestral relationship with Eq (13)*
            if $\lambda_{\mathsf{s}}(j) \neq \lambda_{\mathsf{c}}(l)$ **then**
                ▷ *update ancestry and propagate partial tree rule*
                if update_ancestry$(1, \lambda_{\mathsf{s}}(j), \lambda_{\mathsf{c}}(l))$ = **False then**
                    return False
▷ *propagate SSM phasing with Eq (15)*
for $j \leftarrow 0, 1, \ldots, J-1$ **do**
    for $l \leftarrow 0, 1, \ldots, L-1$ **do**
        if $\mathcal{M}(j, l) = 0$ and $Z(\lambda_{\mathsf{s}}(j), \lambda_{\mathsf{c}}(l)) = 1$ and $\sigma_{\mathsf{s}}(j) = \sigma_{\mathsf{c}}(l)$ **then**
            if $\pi_{\mathsf{s}}(j) = -1$ **then**
                $\pi_{\mathsf{s}}(j) = \rho(\pi_{\mathsf{c}}(l))$
            else if $\pi_{\mathsf{s}}(j) = \pi_{\mathsf{c}}(l)$ **then**
                return False
▷ *propagate SSM phasing with Eq (8)*
for $j \leftarrow 0, 1, \ldots, J-1$ **do**
    for $\alpha \leftarrow A, B$ **do**
        if $\sum_{k^* \in \mathcal{A}(\lambda_{\mathsf{s}}(j))} \Delta C_\alpha(\sigma_{\mathsf{s}}(j), k^*) + \Delta C_\alpha(\sigma_{\mathsf{s}}(j), \lambda_{\mathsf{s}}(j)) = -1$ **then**
            if $\pi_{\mathsf{s}}(j) = -1$ **then**
                $\pi_{\mathsf{s}}(j) = \rho(\alpha)$
            else if $\pi_{\mathsf{s}}(j) = \alpha$ **then**
                return False
return True

Algorithm I propagate_absent_relationships()

Input: (global variables)

Output: whether ancestral relationships can be updated in order to satisfy equivalence and lost allele constraints

▷ *propagate equivalence rules*
for $j \leftarrow 0, 1, \ldots, J-1$ **do**
  for $l \leftarrow 0, 1, \ldots, L-1$ **do**
    ▷ *update ancestral relationship following Eq (16)*
    if $\sigma_{\mathsf{s}}(j) = \sigma_{\mathsf{c}}(l)$ and $\pi_{\mathsf{s}}(j) = \pi_{\mathsf{c}}(l)$ and $\mathcal{M}(j, l) = 0$ and $\lambda_{\mathsf{s}}(j) < \lambda_{\mathsf{c}}(l)$ **then**
      if update_ancestry$(0, \lambda_{\mathsf{s}}(j), \lambda_{\mathsf{c}}(l))$ = **False then**
        return False
    ▷ *update ancestral relationship following Eq (18)*
    else if $\sigma_{\mathsf{s}}(j) = \sigma_{\mathsf{c}}(l)$ and $\sum_{k^* \in \mathcal{A}(\lambda_{\mathsf{c}}(l))} \Delta C_{\pi_{\mathsf{c}}(l)}(\sigma_{\mathsf{c}}(l), k^*) = -1$ and $\mathcal{M}(j, l) = 0$ and $\lambda_{\mathsf{s}}(j) < \lambda_{\mathsf{c}}(l)$ **then**
      if update_ancestry$(0, \lambda_{\mathsf{s}}(j), \lambda_{\mathsf{c}}(l))$ = **False then**
        return False
    ▷ *update ancestral relationship following Eq (17)*
    else
      for $l' \leftarrow 0, 1, \ldots, L-1$ with $l' \neq l$ **do**
        if $(\lambda_{\mathsf{c}}(l') = \lambda_{\mathsf{c}}(l)$ or $\lambda_{\mathsf{c}}(l') \in \mathcal{D}(\lambda_{\mathsf{c}}(l)))$ and $\sigma_{\mathsf{s}}(j) = \sigma_{\mathsf{c}}(l) = \sigma_{\mathsf{c}}(l')$ and $\pi_{\mathsf{c}}(l) = \rho(\pi_{\mathsf{c}}(l'))$ and $\mathcal{M}(j, l) = 0$ and $\mathcal{M}(j, l') = 0$ and $\lambda_{\mathsf{s}}(j) < \lambda_{\mathsf{c}}(l)$ **then**
          if update_ancestry$(0, \lambda_{\mathsf{s}}(j), \lambda_{\mathsf{c}}(l))$ = **False then**
            return False
▷ *propagate lost allele rule*
for $l \leftarrow 0, 1, \ldots, L-2$ **do**
  for $l' \leftarrow l+1, l+2, \ldots, L-1$ **do**
    ▷ *update ancestral relationship following Eq (4)*
    if $\sigma_{\mathsf{c}}(l) = \sigma_{\mathsf{c}}(l')$ and $\pi_{\mathsf{c}}(l) = \pi_{\mathsf{c}}(l')$ and $\lambda_{\mathsf{c}}(l) \neq \lambda_{\mathsf{c}}(l')$ and $\Delta C_{\pi_{\mathsf{c}}(l)}(\sigma_{\mathsf{c}}(l), \lambda_{\mathsf{c}}(l)) = -1$ and $\Delta C_{\pi_{\mathsf{c}}(l')}(\sigma_{\mathsf{c}}(l'), \lambda_{\mathsf{c}}(l')) = -1$ **then**
      if $\lambda_{\mathsf{c}}(l) < \lambda_{\mathsf{c}}(l')$ **then**
        if update_ancestry$(0, \lambda_{\mathsf{c}}(l), \lambda_{\mathsf{c}}(l'))$ = **False then**
          return False
      else
        if update_ancestry$(0, \lambda_{\mathsf{c}}(l'), \lambda_{\mathsf{c}}(l))$ = **False then**
          return False
  for $j \leftarrow 0, 1, \ldots, J-1$ **do**
    ▷ *update ancestral relationship following Eq (6)*
    if $\pi_{\mathsf{s}}(j) = \pi_{\mathsf{c}}(l)$ and $\sigma_{\mathsf{s}}(j) = \sigma_{\mathsf{c}}(l)$ and $\lambda_{\mathsf{s}}(j) > \lambda_{\mathsf{c}}(l)$ and $\Delta C_{\pi_{\mathsf{c}}(l)}(\sigma_{\mathsf{c}}(l), \lambda_{\mathsf{c}}(l)) = -1$ **then**
      if update_ancestry$(0, \lambda_{\mathsf{c}}(l), \lambda_{\mathsf{c}}(j))$ = **False then**
        return False
    ▷ *update ancestral relationship following Eq (7)*
    else if $\sigma_{\mathsf{s}}(j) = \sigma_{\mathsf{c}}(l)$ and $\lambda_{\mathsf{s}}(j) > \lambda_{\mathsf{c}}(l)$ and $\Delta C_{\pi_{\mathsf{c}}(l)}(\sigma_{\mathsf{c}}(l), \lambda_{\mathsf{c}}(l)) = -1$ and $\sum_{k^* \in \mathcal{A}(\lambda_{\mathsf{c}}(l))} \Delta C_{\rho(\pi_{\mathsf{c}}(l))}(\sigma_{\mathsf{c}}(l), k^*) + \Delta C_{\rho(\pi_{\mathsf{c}}(l))}(\sigma_{\mathsf{c}}(l), \lambda_{\mathsf{c}}(l)) = -1$ **then**
      if update_ancestry$(0, \lambda_{\mathsf{c}}(l), \lambda_{\mathsf{c}}(j))$ = **False then**
        return False
return True

V Details on results

V.1 Simulating noise-free subclonal reconstructions

To simulate subclonal reconstructions, we first define parameters controlling the simulated data:

- K: number of subclones including the germline
- N: number of tumor samples
- J: number of SSMs
- L: number of CNAs
- I: number of genomic segments

We then generate simulated data using the following procedure:

1. Generate the tree structure. For each subclone k, with $k \in \{1, 2, ..., K-1\}$, sample a parent $\mathcal{P}(k)$. We extend the previous subclone (i. e., $\mathcal{P}(k) = k-1$) with probability $\mu = 0.75$, and otherwise sample $\mathcal{P}(k)$ from the discrete Uniform$(0, k-1)$ distribution.
2. Generate the population frequencies $\eta(k, n)$ for each population k in each tumor sample n, with $n \in \{0, 1, ..., N-1\}$. These values were sampled for each n as $\{\eta(0, n), \eta(1, n), \ldots, \eta(K-1, n)\} \sim \text{Dirichlet}(1, \ldots, 1)$. Thus, we have $\sum_{k=0}^{K-1} \eta(k, n) = 1$ for each sample n.
3. Compute the subclonal frequencies $\phi(k, n)$ for each subclone k in each tumor sample n using the tree structure and $\eta(k, n)$ values. We have
$$\phi(k, n) = \sum_{k'=0}^{K-1} \eta(k', n) \mathbb{1}_{k'=k \text{ or clone } k' \text{ is a descendant of clone } k}.$$
4. Assign the J SSMs to subclones. To ensure every subclone has at least one SSM, set the subclones of the first $K-1$ SSMs $\lambda_{\mathsf{s}}(0), \lambda_{\mathsf{s}}(1), \ldots, \lambda_{\mathsf{s}}(K-1)$ to $1, 2, ..., K-1$. To assign the remaining $J-K+1$ SSMs, sample subclonal weights from the unit Dirichlet, then sample assignments from the categorical distribution using these weights.
5. Segment the genome into I segments by sampling from Dirichlet$(1, \ldots, 1)$.
6. Generate L CNAs by assigning each event l to a subclone $\lambda_{\mathsf{c}}(l) \in \{1, 2, \ldots, K-1\}$, segment $\sigma_{\mathsf{c}}(l) \in \{0, 1, \ldots, I-1\}$, and phase $\pi_{\mathsf{c}}(l) \in \{A, B\}$. Each assignment is sampled from Dirichlet$(5, \ldots, 5)$ with the appropriate number of dimensions. Subsequently, a direction $d(l) \in \{\text{gain}, \text{loss}\}$ is sampled for every doublet $(\sigma_{\mathsf{c}}(l), \pi_{\mathsf{c}}(l))$, such that all CNAs with the same segment i and the same phase α have the same direction. Moreover, deletions are permitted only once on a given tree branch for a given segment and phase.
7. If the direction $d(l) = \text{gain}$, then the allele gain $g(l)$ is sampled such that $g(l) \sim \text{ceil}(\text{Exponential}(\lambda = 1.5))$.
8. If the direction $d(l) = \text{loss}$, then the allele loss must necessarily be 1, since two CNA events may never have the same segment and phase with opposite directions. This implies that at most one allele can ever be lost.

Table B. Simulated data parameters.

parameter	description	value
K	number of subclones	5, 20, 50
N	number of tissue samples	1, 2, . . ., 20
T	read depth	50x
$J(M)$	number of SSMs	5, 20, 50, 200
$L(C)$	number of CNAs	10, 20, 40
$I(H)$	number of genomic segments	10, 20, 40

The parameter name in brackets gives the name of the parameter in the simulation script. For datasets without CNAs, we generated a total of one SSM per subclone. For datasets with CNAs, we generated 200 SSMs for each dataset. Assignment of SSMs was performed randomly so that every subclone had a variable number of SSM, but received at least one. Code used to generate the simulated data is available at https://github.com/morrislab/pearsim.

9. Sample the timing and phase for each SSM j. SSM phasing $\pi_s(j) \in \{A, B\}$ is sampled from a Dirichlet, such that $\pi_s(j) \sim \text{Dirichlet}(5, 5)$. This phasing is rejected and resampled if the given allele and segment has already been deleted in the SSM's subclone, either in the subclone itself or an ancestor. Subsequently, the SSM's timing $t(j)$ is sampled if a CNA has occurred for the same segment and allele in the SSM's subclone, with $t(j) \in \{\text{before CNA}, \text{after CNA}\}$, and $t \sim \text{Dirichlet}(5, 5)$.

Simulated data parameters are listed in Table B.

V.2 Estimating subclonal frequencies from read counts

Each time when generating count data from the 600 noise-free subclonal reconstructions without CNAs from Section "Simulated data without noise" in the main text, we used a different parameter combination of the number V of SSMs per subclone, selected from 1, 10, and 100, and the total read count Y per SSM, selected from 30, 100, and 300. To generate variant count data for a particular subclone k in sample n with V SSMs, we drew a vector $X \in \{0, \ldots, Y\}^V$ of variant read counts from a Binomial distribution with a total number of Y reads per SSM and a success probability $p = \phi(k, n)/2$, where $\phi(k, n)$ is the noise-free subclonal frequency of the subclone. For each SSM v, with $0 \leq v < V$, we computed its VAF as $X(v)/Y$ and built the estimated subclonal frequency as the mean of the VAFs of its SSMs multiplied by 2, i. e., assuming no copy number influence, while ensuring that the frequency is at most 1. We grouped all data by effective read depth, which is the number of SSMs per subclone multiplied by the number of total reads per SSM. This leads to seven groups with an effective read depth of 30, 100, 300, 1000, 3000, 10000, and 30000, with 1200 subclonal reconstructions with an effective read depth of 300 and 3000, and 600 for each of the other depths.

V.3 Computing the recall and percentage of false positives on the noisy dataset

Because the simulated noise can change the ordering of the subclones, i. e., when a subclone k that had lower average subclonal frequency than a subclone k' in the noise-free data has now a higher frequency, we adapted the order of the subclones in the subMAR of the noisy datasets according to the order in the noise-free MAR. This can lead to values in the lower left triangle of the ancestry matrix Z being different from -1, i. e., an absent relationship. Hence, we computed the recall and the percentage of false positives, by considering all entries $Z(k, k')$ with $k, k' > 0$ and $k \neq k'$.

V.4 Preprocessing of TRACERx data

We worked with the TRACERx data provided in Tables S3 and S7 in the Supplementary Appendix 2 of the work of Jamal-Hanjani et al. [7]. Table S3 contains mutation clusters (column *PyClonePhyloCluster*) and their cancer cellular fraction (CCF, column *PyClonePhyloCCF*) computed by PyClone [8] for 100 patients. After different filtering steps, the authors arrive at 91 patients in Table S7. By avoiding evolutionary conflicts posed by the pigeonhole principle [9] and the crossing rule, and by considering copy number errors, the authors discarded some of the clusters to arrive at a set of mostly consistent clusters (column *TreeClusters* in Table S7) they used to build phylogenetic trees with CITUP [10]. Due to erroneous copy number corrections and a high number of clusters, the authors built manual trees for six patients.

We applied the basic version of SubMARine and used the mostly consistent mutation clusters as subclones and their CCF as subclonal frequencies. Because we did not consider CNAs, we excluded the three datasets with erroneous copy number corrections.

V.5 Using SubMARine with raw and adapted Gundem et al. CCFs

The raw and adapted CCFs of Gundem et al. [11] can be found in their Supplementary Table 3 with the name "Subclones". This table contains the raw CCFs for each of patient starting from column D "CCF in A". Note that the table for patient A17 contains a small error. The frequency of the blue subclone with ID 4 should be 0 in sample E and 1 in sample F as can be read in their description of patient A17 in their supplement (Section 4e) and as can be seen in their Fig 2. The adapted CCFs can be found in their Supplementary Table 3 in column "CCF values". Note that for the light blue subclone with ID 20 in patient A22, the authors work with a CCF of 0.39 in sample F in order to satisfy the pigeonhole principle as described in their supplementary Section 4e. Also note that for patient A21, the authors report a colorless subclone with ID 18 that they do not use in their reconstructions, so we decided to ignore it.

In order to compute subclonal frequencies, we multiplied the CCFs with the purity values per sample, which can be found in Supplementary Table 1 of Gundem et al. with the name "Samples" in column "Tumour cellularity".

V.6 Using SubMARine in extended mode on Gundem et al. data

In order to run SubMARine in extended mode on the Gundem et al. data, we first cleaned the publicly available data before parsing it for PhyloWGS [12]. We then ran PhyloWGS for the five patients A10, A12, A17, A24 and A34 and chose the clone tree with the highest likelihood. Afterwards, we computed a mapping between the subclones of PhyloWGS and Gundem et al. and finally, parsed PhyloWGS' output to use it for SubMARine.

V.6.1 Cleaning data

Cleaning SNV data. The SNVs of the coding regions for each patient can be found in Table "substitutions" of the Supplementary Data of Gundem et al. The initial table contained 7699 rows. We removed identical rows, which resulted in 7673 rows.

Patient A32 had multiple entries in sample C for one SNV (chromosome 8, position 24190214) we did not understand: Four entries existed, indicating a mutation from C to T and C to A twice with identical VAFs for all four rows. Hence, we removed this mutation for patient A32 in all samples.

For patient A21, we removed the SNV on chromosome 15 at position 102294332 because it had no coverage at all in WGS and was not deep sequenced. Also, the SNV on chromosome 21 at position 10181201 had very low coverage (max. 6) in all nine samples, in one sample even no coverage. Hence, we removed it as well.

For patient A17, the Dirichlet process applied by Gundem et al. identified among others the two clusters 4 and 7. The validation through deep sequencing showed that these clusters could be split up into two. Hence, we changed the cluster or subclone assignment for some SNVs of patient A17:

- mutations previously assigned to cluster 4 that have a WGS VAF of 0 in sample D or that failed validation, were assigned to a new cluster with ID 6
- mutations previously assigned to cluster 7 that have a WGS VAF greater than 0 in sample D were assigned to a new cluster with ID 8

For the 14 samples across the patients that were not subject to WGS but only to deep sequencing, only a subset of the SNVs per patient were sequenced. Thus, we removed these samples.

Cleaning indel data. The indels of the coding regions for each patient can be found in Table "indels" of the Supplementary Data of Gundem et al. The initial table contained 611 rows. Removing identical rows resulted in 534 rows. After removing samples that were not whole genome sequenced the table contained 438 rows. No further cleaning was necessary.

Cleaning CNA data. The CNAs across the whole genome for each patient can be found in Table "copy_number" of the Supplementary Data of Gundem et al. The initial table contained 15742 rows and 68 columns in the Battenberg [9] format. Because the PhyloWGS parser, which we used later, works only with the first 14 columns of this format, we discarded the other columns.

24 rows contained frequency values for the fields "frac1_A" and "frac2_A" that were smaller than 0 or greater than 1. Hence, we removed these rows.

Another 24 rows contained major copy numbers that were smaller than their minor copy numbers. Thus, we swapped these values.

For patients A12, A22, A24, A31, A32, and A34 on chromosome X, some copy number calls overlapped within the same sample. This is why we removed all calls on chromosome X for these six patients.

V.6.2 Parsing data for PhyloWGS

To parse the mutational data for PhyloWGS, we used the PhyloWGS input parser[1]. In order to use this parser, we first had to bring the data in the right format. For this purpose, we merged the SNVs and indel calls for each patient and sample into a VCF file. If a mutation was successfully validated by deep sequencing, we used the VAF and read depth information from the validation to compute the number of reference and variant alleles for the VCF file. If the validation failed, we set the number of variant alleles to 0. If the mutation was not validated through deep sequencing, we computed the number of its reference and variant alleles from the WGS VAF and read depth.

To create preliminary CNA input for PhyloWGS, we used the script *parse_cnvs.py* with the option for the Battenberg algorithm and cellularity values according to Gundem et al.'s Supplementary Table 1 "Samples" for each patient and each sample. Afterwards, we created the final input files with the script *create_phylowgs_inputs.py* with the sex of the patients set to male and the standard option to remove all regions in the genome for which either no copy number information is provided or for which multiple CNAs overlap. When removing these regions, also all SSMs that are present in these regions were excluded. For some patients, this resulted in losing more than half of their SSMs. Hence, we decided to apply PhyloWGS only on those patients for which at least half of their SSMs were still present. These are patients A10, A12, A17, A24 and A34.

V.6.3 Running PhyloWGS and choosing a clone tree

We ran PhyloWGS on the data for patients A10, A12, A17, A24 and A34 with four MCMC chains and the default number burnin and MCMC samples. Afterwards, we chose the clone tree with the highest likelihood for each patient.

[1] https://github.com/morrislab/phylowgs/tree/master/parser

V.6.4 Computing a mapping between subclones of PhyloWGS and Gundem et al.

Because PhyloWGS built its own subclones based only on a subset of the data used by Gundem et al., the subclones between PhyloWGS and Gundem et al. are not identical. In order to be able to compare the constructed clone trees, we computed a mapping between the subclones, using information on SNV assignments and subclonal frequencies.

For the SNV assignment comparison, we compared the number of equally and differently assigned mutations for each PhyloWGS subclone with each Gundem et al. subclone to which at least one of the coding mutations used by PhyloWGS was assigned. We noted a mapping between a subclone pair if the number of equal mutations was higher than the number of differing mutations (S4 Table).

For the subclonal frequency comparison, we first converted the raw CCF values by Gundem et al. to subclonal frequencies as described in Section V.6. Then we highlighted all those subclone and sample pairs in the subclonal frequency matrix for which Gundem et al. indicate the existence of the subclone in the sample (Supplementary Table 3 "Subclones" of Gundem et al., column "samples containing", and S4 Table). Next, for the PhyloWGS subclonal frequency matrix, we highlighted all those subclone and sample pairs in which the subclone has a frequency of at least 0.1. We then assigned PhyloWGS subclones to Gundem et al. subclones based by eye and minimal pairwise distance.

Afterwards, we combined the SNV assignment and frequency mapping to a final mapping, shown in S4 Table. Note that not all PhyloWGS subclones could be mapped to a Gundem et al. subclone and not all Gundem et al. subclones got a PhyloWGS subclone assigned.

V.6.5 Parsing data for SubMARine

Finally, we parsed PhyloWGS' output to provide the input needed for extended SubMARine. Because PhyloWGS does not phase SSMs in regions with copy number changes, we computed the phasing with the highest likelihood to derive CNA influence on SSM copy numbers. In order to do so, for each SSM if it was either assigned to a subclone ancestral to a subclone containing a CNA in the same genome segment or if it was assigned to a subclone that had also assigned a CNA in the same segment, we did the following:

- get major and minor copy number of the CNA
- assuming the SSM was assigned to the major allele, which corresponds to our allele A, compute the likelihood as follows:
  - compute the copy number of the variant allele and of the reference allele for each sample
  - compute the expected proportion of reads containing the reference allele from these copy numbers for each sample
  - compute the binomial likelihood of observing the given number of reference reads based on the total number of reads and the expected proportion for each sample
  - sum up the likelihoods for all samples
- repeat computing the likelihood assuming the SSM was assigned to the minor allele, which corresponds to our allele B
- choose the highest likelihood and if the corresponding major or minor copy number is not 1, hence indicates a copy number change, phase the SSM to the corresponding allele and indicate that the CNA influences the copy number of the SSM

References

1. Sundermann LK. Lineage-Based Subclonal Reconstruction of Cancer Samples [dissertation]. Bielefeld University; 2019. Available from: https://pub.uni-bielefeld.de/record/2935248.

2. El-Kebir M, Oesper L, Acheson-Field H, Raphael BJ. Reconstruction of clonal trees and tumor composition from multi-sample sequencing data. Bioinformatics. 2015;31(12):i62–i70. https://doi.org/10.1093/bioinformatics/btv261.

3. El-Kebir M, Satas G, Oesper L, Raphael BJ. Inferring the mutational history of a tumor using multi-state perfect phylogeny mixtures. Cell Systems. 2016;3(1):43–53. https://doi.org/10.1016/j.cels.2016.07.004.

4. Popic V, Salari R, Hajirasouliha I, Kashef-Haghighi D, West RB, Batzoglou S. Fast and scalable inference of multi-sample cancer lineages. Genome Biology. 2015;16(1). https://doi.org/10.1186/s13059-015-0647-8.

5. Jiao W, Vembu S, Deshwar AG, Stein L, Morris Q. Inferring clonal evolution of tumors from single nucleotide somatic mutations. BMC Bioinformatics. 2014;15(1):35. https://doi.org/10.1186/1471-2105-15-35.

6. Qi Y, Pradhan D, El-Kebir M. Implications of non-uniqueness in phylogenetic deconvolution of bulk DNA samples of tumors. Algorithms for Molecular Biology. 2019;14(1):19. https://doi.org/10.1186/s13015-019-0155-6.

7. Jamal-Hanjani M, Wilson GA, McGranahan N, Birkbak NJ, Watkins TB, Veeriah S, et al. Tracking the evolution of non–small-cell lung cancer. New England Journal of Medicine. 2017;376(22):2109–2121. https://doi.org/10.1056/NEJMoa1616288

8. Roth A, Khattra J, Yap D, Wan A, Laks E, Biele J, et al. PyClone: statistical inference of clonal population structure in cancer. Nature Methods. 2014;11(4):396. https://doi.org/10.1038/nmeth.2883.

9. Nik-Zainal S, Van Loo P, Wedge DC, Alexandrov LB, Greenman CD, Lau KW, et al. The life history of 21 breast cancers. Cell. 2012;149(5):994–1007. https://doi.org/10.1016/j.cell.2012.04.023.

10. Malikic S, McPherson AW, Donmez N, Sahinalp CS. Clonality inference in multiple tumor samples using phylogeny. Bioinformatics. 2015;31(9):1349–1356. https://doi.org/10.1093/bioinformatics/btv003.

11. Gundem G, Van Loo P, Kremeyer B, Alexandrov LB, Tubio JM, Papaemmanuil E, et al. The evolutionary history of lethal metastatic prostate cancer. Nature. 2015;520(7547):353–357. https://doi.org/10.1038/nature14347.

12. Deshwar AG, Vembu S, Yung CK, Jang GH, Stein L, Morris Q. PhyloWGS: reconstructing subclonal composition and evolution from whole-genome sequencing of tumors. Genome Biology. 2015;16(1):35. https://doi.org/10.1186/s13059-015-0602-8.
