# Supplementary material for: Reconstructing tumor evolutionary histories and clone trees in polynomial-time with SubMARine
Source: PLoS Comput Biol. 2021 Jan 19;17(1):e1008400. doi: 10.1371/journal.pcbi.1008400 (PMC7845980; doi:10.1371/journal.pcbi.1008400)
Supplement: Attachment — Submitted filename: RECOMB-CCB_responseToReviews.pdf [file pcbi.1008400.s032.pdf]

Reviews - RECOMB-CCB

---------------------- REVIEW 1 --------------------

SUBMISSION: 475
TITLE: Reconstructing Tumor Evolutionary Histories and Clone Trees in Polynomial-time with SubMARine
AUTHORS: Linda K. Sundermann, Jeff Wintersinger, Gunnar Rätsch, Jens Stoye and Quaid Morris

----------- Overall evaluation -----------

SCORE: 0 (borderline paper)
----- TEXT:
Summary:
In this work the authors present a particular representation of a set of clone trees, which they call a partial clone tree. They then describe a particular instance of a partial clone tree called the Maximally-Constrained Ancestral Reconstruction (MAR). Finally, they describe a polynomial time algorithm, SubMARine, that produces an approximation to the MAR called the subMAR. As several studies have recently shown that a large number of clonal trees may be consistent with an observed dataset (Pradhan and El-Kebir, 2018 and Tomlinson and Oesper, 2019), a method that is able to accurately represent these large sets of trees without enumerating all of them is very appealing. However, despite the potential impact of this work, there are a number of significant limitations to it, in its current form.

Thank-you for your helpful feedback and for pointing out the importance of our work

Major Comments:

1. Assuming that subclonal frequencies are measured precisely is unrealistic on real data. While I appreciate that the authors do note that they were still able run on real data, inclusion of more information on how they expect that this assumption will affect the produced results would be very helpful. Perhaps adding some noise to the simulations and analyzing how this affects the results.

We do not believe including noise in our simulations would be any more informative than the results we already report on the real data, where our method was able to reconstruct trees for approximately half of the tumors.

In particular, for a specified observation noise process, and many tree-generation processes, in many cases, one can derive analytically how often the sum constraint would be violated for the correct tree. Doing that analytical computation is beyond the scope of this work but would represent an interesting manuscript in its own right.

Instead, to address this concern, we have introduced a parameter to account for noise. This parameter is the maximum allowed violation to the sum constraint. This parameter can be set using a bounded binary search based strategy to determine how much violation is required before a non-null subMAR can be found for the dataset. This new addition to the algorithm is described in Sections 3.2 and S4.3.

2. There are a number of aspects of the organization of sections 3 and 4 that obscure the technical content being presented. Here are a few suggestions.
a. The name partial clone tree is misleading, since it doesn't actually represent a tree, but rather a set of potential trees. Also, as written, both a partial clone tree and the MAR are defined to be the solution to the basic maximally-constrained ancestral reconstruction problem. I don't think this is what is meant. You might fix this by defining the partial clone tree as a subset of pairwise ancestral relationships present in all valid clone trees, and the MAR as the maximum sized such set. As it stands, the word max is never actually used when defining the MAR. Furthermore, Fig 1 would be improved by adding an example of a partial clone tree and the MAR. This would help delineate the difference between these two.

*"Partial clone tree" is short for "*partially-defined *clone tree". We have attempted to make that clear in the manuscript by modifying text in the contributions section. The relevant text now reads:*

> *Here we introduce and formalize the notion of a partially-defined clone tree, or partial clone tree for short.… A partial clone tree is not a tree itself, but it implicitly defines a set of clone trees, i.e., all those trees that (i) are consistent with the ancestral relationships defined in the partial clone tree and (ii) select their parents from the possible parent set.*

All other suggestions were implemented.

b. The authors state that a partial clone tree cannot be represented as a DAG when Z(k,k') = 1, and therefore Gabow-Myers cannot be used. It is not clear why this is true (especially since the authors represent a partial clone tree as a DAG in figure 1d). Please add additional clarification.

Thank you for pointing this lack of clarity out. The DAG in Figure 1d) is indeed a representation of all the trees that complete the partial clone tree, in the following way: all spanning trees of the

DAG complete the partial clone tree. However, it is not always the case that there is a DAG which fully represents a partial clone tree such that every spanning tree in the DAG completes the ancestry matrix Z. This is why we cannot use Gabow-Myers. We clarified this now and added a supplementary section (Section S3.1) with an example.

c. SubMARine is described as a major contribution of this work, but is only described in detail in the appendix. Additional details (perhaps pseudo code) should be added to the main text, especially since this work appears aimed at a computational audience. In particular, the section describing the SubMARine approach could be made much more crisp. As it stands a number of claims are stated (always converges, order doesn't matter) that would be made much stronger if addressed in a more rigorous manner.

We rewrote the section describing the SubMARine approach and included a functional description of the algorithm as well as an overview over all used inference rules derived from the validity constraints. We now address our theoretical claims in an easier to understand manner.

d. The explanation of definite children seems essential to the approach. Inclusion of a demonstration of how this is determined for the example in Figure 1, just from the observed frequencies (or at least referencing back to this figure) would be helpful.

When defining definite children and the generalized sum constraint, we now reference back to Figure 1.

3. Inclusion of results that show how the output from SubMARine is used to identify problematic subclones for the TRACERx data would be useful. It seems odd that the actual output for any specific patient is not included.

We now provide the actual output for every patient in an additional Excel spreadsheet (Appendix 2, Table S4). The information which subclones conflict with the sum constraint is also included. Furthermore, we updated SubMARine so that its log file output now explicitly points to these conflicting subclones.

Minor Comments:

1. Results section - it is odd to use the phrase “thus without and with CNAs”. It is much more readable to say “thus with and without CNAs”.

We chose this order because “without” refers to the basic clone tree reconstruction problem and “with” to the extended one which are mentioned in this order in the first part of the sentence.

2. Simulated data - typo “70seconds” with no space.

Corrected.

---------------------- REVIEW 2 --------------------

SUBMISSION: 475
TITLE: Reconstructing Tumor Evolutionary Histories and Clone Trees in Polynomial-time with SubMARine
AUTHORS: Linda K. Sundermann, Jeff Wintersinger, Gunnar Rätsch, Jens Stoye and Quaid Morris

----------- Overall evaluation -----------

SCORE: 2 (accept)
----- TEXT:
This paper considers a timely problem in tumor phylogeny inference from bulk data, where many equally plausible phylogenies exist for the same input data. The authors propose to summarize the solution space of trees through a Maximally Ambiguous Reconstruction (MAR), which is the set of ancestral relationships between pairs of mutations present in all solution trees (each ancestral pair is designated as either present in all trees or partially present). By definition the MAR is unique. The authors provide a heuristic for approximately reconstructing the MAR, in both the simple setting of only SNVs as well as with additional CNAs. This is good work, and a substantial improvement from the original RECOMB paper, which I previously reviewed (Reviewer 1). My previous comments have been satisfactorily addressed. I only have minor comments.

Thank-you for your helpful comments in your first review; they helped improve the paper considerably.

Minor comments:

* Abstract: "...that the defined pairwise relationship." This sentence needs editing.

Done.

* Fig 1d. What are the dashed edges?

We've added a description of the edges to the figure legend.

* Page 4: an directed edge => a directed edge

Done.

* Page 4: include reference to Gabow-Myers.

Done.

* Discussion: Good to state that hardness of MAR problem remains open, as well as approximation factor of the subMAR.

We have added the following text to the discussion:

> *There are a number of unanswered theoretical questions raised by this work. First, it is unclear what the hardness of the MAR reconstruction problem is. Because a MAR only exists if there is at least one valid clone tree solution, it seems likely that MAR reconstruction is at least as hard as the problem of finding a single clone tree solution. However, it is not clear whether this hardness changes under the assumption that a valid clone tree exists. Neither of these two questions are addressed by SubMARine. Also SubMARine approximates the MAR but provides no guarantees about its approximate factor. It would be useful to provide such guarantees, if they exist. Or perhaps a different algorithm to generate subMARs can provide them.*

----------------------- REVIEW 3 ---------------------

SUBMISSION: 475
TITLE: Reconstructing Tumor Evolutionary Histories and Clone Trees in Polynomial-time with SubMARine
AUTHORS: Linda K. Sundermann, Jeff Wintersinger, Gunnar Rätsch, Jens Stoye and Quaid Morris

----------- Overall evaluation -----------

SCORE: 1 (weak accept)
----- TEXT:
This manuscript addresses the relevant problem of phylogeny inference in sub-clonal evolution of tumors. The authors do so introducing a new approach 'Maximally-constrained Ancestral

Reconstruction (MAR)' for partial clone trees that defines a subset of the pairwise ancestral links in a clone tree that is able to summarize the entire field of all solutions of clone trees that fit the input data. The authors also present 'SubMARine' an algorithm to efficiently generate an approximation of MAR named 'subMAR'. The manuscript describes the implementation of 'SubMARine' using sub-clonal frequencies, somatic single nucleotide variants (SNVs) and somatic copy number variations (CNVs). Last, the approach was applied using simulated data and data of lung cancer donor from TRACERx study. Although the manuscript extensively describes interesting conceptual hypothesizes for inferring sub-clonal trees and their algorithmic solutions, this reviewer found some issues that, if addressed, the manuscript will improve its quality to subsequent publishing.

Thank-you for your feedback, we have addressed all of your concerns.

Major:

1- The authors clearly and explicitly point out two aims for the work: 1) an efficient algorithm for clone trees with many sub-clones and 2) efficiently capturing uncertainty in the clone trees. Thus this reviewer understand the scope of the work on regard to the starting point of the inputs but, a paper that address the study of tumor sub-clonality have to talk about the major factors in obtaining the related data such “tumor purity” and “sequencing coverage” and this manuscript does not mention it at all. In sections like introduction, background and discussion it is crucial to clarify how those factors affect the tumor sub-clones trees and where this proposal stands in relation to those mentioned factors. That will put this paper in better context for researchers of several necessities in this challenge of understanding tumor sub-clonality.

We have added text to the introduction, background, and discussion section to address these concerns. We now acknowledge that there are substantial challenges introduced in subclonal reconstruction by inaccuracies in the estimates of the cellular frequencies of mutations and the copy number reconstructions. These challenges can arise when tumor purity and sequencing coverage are low. This manuscript, however, is primarily focused on the additional challenge of characterizing the space of clone tree solution even under ideal conditions of nearly noise-free input data. For example, we have added the following text to the background section:

> *“The accuracy of the cellular frequency estimates, CNA reconstructions, and subclonal groupings depends heavily on the sequencing depth, degree of aneuploidy, and purity of the samples. However, even under the best of conditions, when there is high accuracy in all of these, there remain substantial challenges in clone tree reconstruction.”*

We have also extended SubMARine so allow for some noise in the estimates of subclonal frequency when performing reconstructions by introducing a noise buffer.

2- It is always hard to follow description of algorithms and data structures. In that sense the manuscript has illustrative figure but they could be more integrated with the text narrative.
- In section 3 the figure can be enhanced to reflect the rationale on creating the 'Z' matrix and therefore it will be more self-explanatory and more connected to the text.

Done.

- The section 3.2 needs a support figure for the logic of text as well as the section 4 where I suggest to use figure S3 also enhanced to better connected with the text.

New figures for section 3.2 and section 4 are included. Caption of previous Figure S3 is adapted to better describe the flow of SubMARine.

3- For this type of papers, it is informative and very useful to get benchmarks of the new approach respect to others methods getting for example a comparison table of the required input and the outcome trees. The manuscript mentions CITUP was used to infer the clone trees in the same TRACERx data set that subMARine was used. The full comparison of those results is important to evaluate the novelty of this approach.

We now included the full comparison to CITUP in Appendix 2, Table S4 and added a paragraph to our result description where we point out certain aspects of this comparison.

Minor:

1- Organizationally and esthetically the first cite should be reference number 1 and so on following the order of citing. That will help to reduce the cite format of the long list of references in paragraph 3 of introduction.

Done.

2- The citing of section S5.5 comes before of the S5.4, it could be reordered

We specifically chose this order, which might be confusing at first sight for the following reason: Before explaining the extended version of SubMARine in more detail, we briefly state the properties of the extended subMAR, which can formally only be explained after showing the algorithm. However, before explaining the algorithm in some more detail, we have to explain the monotonicity restriction, which we chose to get its own subsection.

3- Figure S10 f), wrong duplication numbering: dupl 3 should be dupl 0

Thanks for spotting this. Is corrected.

4- Section S3.1 first paragraph, typo in the if-then structure sentence: “than” for then (twice)

Corrected.
