# Supplementary material for: Reconstructing tumor evolutionary histories and clone trees in polynomial-time with SubMARine
Source: PLoS Comput Biol. 2021 Jan 19;17(1):e1008400. doi: 10.1371/journal.pcbi.1008400 (PMC7845980; doi:10.1371/journal.pcbi.1008400)
Supplement: Attachment — Submitted filename: PLOSCB_responseToReviews.pdf [file pcbi.1008400.s033.pdf]

Reviews - PLOS CB

Reviewer #1:

This manuscript introduces SubMARine, a method to infer a summary of phylogenetic trees that explain bulk sequencing data of tumors. Basically, given a solution space of trees the maximally-constrained ancestral reconstruction (MAR) designates all ordered pairs (i,j) of mutations as either ancestral (if i is ancestral to j in all trees), not ancestral (if i is not ancestral to j in all trees) or ambiguous (otherwise). Since exhaustive enumeration of the solution is intractable, the authors introduce a relaxation of the MAR, the subMAR, obtained directly from the frequency matrix. The subMAR (just like the MAR) is unique but may contain more ambiguous entries. In addition to the clean problem without CNAs, the authors consider a version of the problem with CNAs. I have two major comments and several minor comments.

Thank-you for your summary!

Major:

1. Please provide a real data application of the extended SuBMARine algorithm.

While simulations are used to assess the performance of their algorithms in both problems settings, the TRACERx non-small-cell lung cancer data is used to assess only the basic version of the problem without CNAs. I would like to see a real application of the extended SubMARine algorithm.

We have now included an application of the extended version of SubMARine to data from Gundem et al., see Section 5.4.

2. Dealing with uncertainty

Compared to the conference version, this manuscript contains an extension of the algorithm to account for uncertainty in the frequency matrix. It would be good to include more methodological details in the main text about this. Moreover, it would be good to assess the performance of SubMARine using simulated data where one accounts for uncertainty in read counts.

We now included more information about the noise buffer in the main text.
We also simulated data accounting for uncertainty in read counts and evaluated SubMARine's performance on it in Section 5.2.

Minor:

* Pseudo code:
- Line 1: As \phi is a matrix, I would not write |\phi| to indicate the number of rows. Simply define \phi to be a K by N matrix.

A good idea. We did this.

- What is Equation 9? (this equation is also referred to several times in the main text).

Equation 9 describes the crossing rule in Section S4.1. We added the information of this supplementary section, so that readers can find the equation easier.

* Author summary: "up o 50" => "up to 50"

Thanks for spotting this typo.

* Line 140, 144, etc. What is t? Isn't the basic clone tree reconstruction problem defined by phi?

The variable t describes the basic clone tree reconstruction problem, which in addition to \phi can also contain SSMs and subclonal CNAs as stated in lines 122-124 (of the new paper version). However in the basic problem, these mutations have no influence on the pairwise ancestral relationships between subclones, which is why they do not have to be considered in Problem 1.

* Line 230. Elaborate on sorting of phi. Do you sort in ascending order of frequencies in first sample? How do you break ties?

We added information in the main text that we sort in decreasing order of the average subclonal frequencies across samples and that we break ties by sorting according to a user-provided subclonal ID.

Reviewer #2:

The authors have significantly improved the manuscript. The methodology is clearly explained and supported by new figures and supplementary materials. The revised version of the paper covers some crucial topics more extensively, such as: 1) The authors have introduced a noise buffer to extend SubMARine to handling some noise in the estimates of subclonal frequency when performing reconstructions; 2) Full comparison of the results with CITUP method.

The changes introduced to the algorithm in this updated version address satisfactorily my concerns in my first round of review. In my opinion the methods and the manuscript have enhanced scientific quality addressing the challenges of clone tree reconstruction.

Thank-you for your helpful and constructive comments in your first review; with them we could improve the paper considerably.
